# In Vivo and In Vitro Models of Hepatocellular Carcinoma: Current Strategies for Translational Modeling

**DOI:** 10.3390/cancers13215583

**Published:** 2021-11-08

**Authors:** Guilherme Ribeiro Romualdo, Kaat Leroy, Cícero Júlio Silva Costa, Gabriel Bacil Prata, Bart Vanderborght, Tereza Cristina da Silva, Luís Fernando Barbisan, Wellington Andraus, Lindsey Devisscher, Niels Olsen Saraiva Câmara, Mathieu Vinken, Bruno Cogliati

**Affiliations:** 1 Department of Pathology, School of Veterinary Medicine and Animal Science, University of São Paulo (USP), São Paulo 05508-270, Brazil; romualdo.gr15@gmail.com (G.R.R.); cicerocosta@usp.br (C.J.S.C.); terezacs678@gmail.com (T.C.d.S.); 2 Department of Structural and Functional Biology, Biosciences Institute, São Paulo State University (UNESP), Botucatu 18618-689, Brazil; gabriel.bacil@unesp.br (G.B.P.); luis.barbisan@unesp.br (L.F.B.); 3 Department of Pathology, Botucatu Medical School, São Paulo State University (UNESP), Botucatu 18618-687, Brazil; 4 Department of Pharmaceutical and Pharmacological Sciences, Vrije Universiteit Brussel, 1090 Brussels, Belgium; Kaat.Leroy@vub.be (K.L.); Mathieu.Vinken@vub.be (M.V.); 5 Gut-Liver Immunopharmacology Unit, Basic and Applied Medical Sciences, Liver Research Center Ghent, Faculty of Medicine and Health Sciences, Ghent University, 9000 Ghent, Belgium; Bart.Vanderborght@ugent.be; 6 Hepatology Research Unit, Internal Medicine and Paediatrics, Liver Research Center Ghent, Faculty of Medicine and Health Sciences, Ghent University, 9000 Ghent, Belgium; LINDSEY.DEVISSCHER@ugent.be; 7 Department of Gastroenterology, Clinics Hospital, School of Medicine, University of São Paulo (HC-FMUSP), São Paulo 05403-000, Brazil; wellingtonandraus@gmail.com; 8 Department of Immunology, Institute of Biomedical Sciences IV, University of São Paulo (USP), São Paulo 05508-000, Brazil; niels@icb.usp.br

**Keywords:** hepatocarcinogenesis, liver cancer, animal model, cell culture, gene mutation, epigenetic alteration, translational research

## Abstract

**Simple Summary:**

Hepatocellular carcinoma (HCC) is a highly incident and deadly malignant neoplasia, and only a few anti-HCC drugs are currently available. Thus, the development of HCC models has become essential for both basic and translational research, improving the understanding of HCC pathophysiology and molecular landscape. The present paper provides a state-of-the-art overview of in vivo and in vitro models used for translational modeling of HCC, focusing on their molecular hallmarks. Our paper depicts the key features, advantages and disadvantages of the main bioassays available, shedding light on standard HCC model choice.

**Abstract:**

Hepatocellular carcinoma (HCC) is the sixth most common cancer worldwide and the third leading cause of cancer-related death globally. HCC is a complex multistep disease and usually emerges in the setting of chronic liver diseases. The molecular pathogenesis of HCC varies according to the etiology, mainly caused by chronic hepatitis B and C virus infections, chronic alcohol consumption, aflatoxin-contaminated food, and non-alcoholic fatty liver disease associated with metabolic syndrome or *diabetes mellitus*. The establishment of HCC models has become essential for both basic and translational research to improve our understanding of the pathophysiology and unravel new molecular drivers of this disease. The ideal model should recapitulate key events observed during hepatocarcinogenesis and HCC progression in view of establishing effective diagnostic and therapeutic strategies to be translated into clinical practice. Despite considerable efforts currently devoted to liver cancer research, only a few anti-HCC drugs are available, and patient prognosis and survival are still poor. The present paper provides a state-of-the-art overview of in vivo and in vitro models used for translational modeling of HCC with a specific focus on their key molecular hallmarks.

## 1. Hepatocellular Carcinoma: Worldwide Trends and Mechanisms

### 1.1. Epidemiology and Contributing Factors

Liver cancers, mainly represented by hepatocellular carcinoma (HCC), accounted for about 840,000 incident cases and 780,000 deaths in 2018 [1]. HCC corresponds to approximately 78% of all hepatobiliary malignancies, being the sixth most incident neoplasm and the third leading cause of cancer-related deaths worldwide [2]. HCC has a poor prognosis, displaying an average survival of 11 months and a survival rate of 49–63%, 19–29%, and 17% after 1, 3, and 5 years of diagnosis, respectively [3,4]. Over 90% of HCC cases occur in a fibrotic or cirrhotic background, which is considered the main risk factor [1,5]. Moreover, populational data on HCC display two important features: geographical and gender disparities. Standardized incidence rates (cases or deaths per 100,000 people) in Asian and African continents are ~2-fold higher than in Europe and North America [1]. While most HCC cases globally are caused by chronic hepatitis B and/or C virus (HBV/HCV) infections (44–56% to HBV and 20–21% to HCV), lifestyle-related risk factors are fast-growing populational attributable factors for this malignancy in western HCC patients. Non-alcoholic fatty liver disease (NAFLD) is closely associated with metabolic syndrome and *diabetes mellitus*, which are independently linked to as many as 16% of HCC cases worldwide [6]. Excessive and chronic alcohol intake leading to alcoholic liver disease (ALD) is another important risk factor associated with 26% of HCC cases, standing only behind HBV infection. In Central Asia and Central Sub-Saharan Africa, HBV and HCV chronic infections are indeed the most prominent risk factors, responsible for 57–60% and 41–50% of all cases, respectively [6]. On the other hand, in Central Europe and North America, ALD and NAFLD-related metabolic syndromes are the most prominent ones, linked to 30–32% and 20–24% of all cases, respectively [6,7]. Since some authors consider chronic viral infections as the most important risk factors for HCC development, HBV/HCV-related HCC attributable fraction may in part explain the geographical disparity feature. Another important epidemiological feature is the marked male disparity (two to three-fold higher in males), whose mechanisms may involve the predominance of risk factors in men and the promoting/protective roles of sex hormones [2]. The influence of dietary factors on HCC emergence is not fully understood, but many epidemiological studies point to a marked protective effect of coffee consumption [8,9].

### 1.2. Hepatocarcinogenesis

Hepatocarcinogenesis represents a complex multistep process in which successively more aberrant monoclonal populations of hepatocytes evolve [10]. The pro-inflammatory and pro-fibrotic microenvironment forms the ideal background for the emergence of numerous human hepatocarcinogenesis-promoting genetic and epigenetic abnormalities [11,12]. Many cancer driver pathways have been repeatedly altered in HCC according to the distinct genotoxic insults and etiologies, allowing the classification of HCC in molecular and/or immune subclasses [13]. To unveil the main molecular alterations involved in HCC, The Cancer Genome Atlas Research Network (TCGA) has performed the first large-scale multi-platform analysis of HCC, including the evaluation of somatic mutations, DNA methylation, gene, protein, and microRNA (miRNA) expressions [14]. Further, Llovet et al. [13] recently segregated HCCs into two major morphological/pathophysiological/molecular phenotypes: proliferation and non-proliferation classes. The proliferation class is more aggressive and poorly differentiated, frequently related to HBV-related etiology. The non-proliferation phenotype is less aggressive, well-to-moderately differentiated, and linked to HCV, alcohol, and NASH-related causes. Telomerase reverse transcriptase (TERT) promoter mutations are the most common mutations in all HCCs analyzed (44%), frequently observed in both phenotypes and in co-occurrence with CDKN2A (p16) hypermethylation (53%), which is more common in the non-proliferation class. Upregulation of TERT and downregulation of CDKN2A enables the immortalization cancer hallmark. The activation of the Wnt/β-catenin pathway, conferring sustained proliferation hallmark, was also frequently featured in both phenotypes, as inactivating tumor suppressor AXIN1 (8%) and activating oncogene CTNNB1 (27%) mutations were observed in proliferation and non-proliferation classes, respectively. HBV-related proliferation class is also associated with the activation of key proliferation pathways, as PI3K–AKT–mTOR, RAS–MAPK, MET, and IGF. TP53 mutations (31%), conferring “evasion of growth suppressors”, “genomic instability and mutation”, and “resistance to cell death” cancer hallmarks, were frequently observed in proliferation class, also in keeping with global DNA hypomethylation signature [13,14,15]. As Wnt/β-catenin and TP53 pathways or TERT are altered in ~77% of HCCs, these dominant molecular drivers are key molecular therapeutic targets and remain undruggable [13,14].

In light of the unknown HCC molecular landscape and the urgent need for novel preventive and therapeutic strategies, the establishment of HCC models has become essential for both basic and translational research. Recently, with the continuous emergence of precision and personalized medicine, standardized and personalized HCC models are warranted. To achieve these requirements, the model should recapitulate key pathophysiological and molecular events observed during hepatocarcinogenesis in view of being effectively translated into clinical practice. Considering the current myriad of HCC models in the literature, we provide a comprehensive overview of the main in vivo and in vitro bioassays applied for HCC modelling, depicting their key molecular hallmarks.

## 2. In Vivo Models of HCC

### 2.1. Syngeneic and Xenograft Mouse Models

Syngeneic and xenograft experimental models are based on the injection or implantation of HCC cell lines or patient-derived xenograft (PDX) in either extrahepatic (heterotopic) or intrahepatic (orthotopic) microenvironments. In the syngeneic mouse models, injection of a murine HCC cell line enables the evaluation of molecular characteristics and tumor growth in a microenvironment of immunocompetent animals [16]. The xenografts mouse models comprise injection of human HCC cells or transplantation of fresh PDX into immunodeficient animals, such as non-obese diabetic/severe combined immunodeficiency (NOD-*scid*) and athymic Balb/c nude mice, delivering a translational model of HCC that recapitulates some of the relevant genetic alterations, i.e., TP53, FGFR1, and KRAS mutations [16,17,18,19]. The *scid*-mutated mice are leucopenic and have a compromised function of B and T lymphocytes, while NOD-*scid* mice feature both impairment of leucocyte activity and diminished activity of natural killer cells and innate immunity, allowing them to be easily grafted [20,21]. To establish a translational model to evaluate HCC, the PDX mouse model underwent improvements, and humanized mice, which will be further reviewed (see 2.5 Humanized mouse models), have been developed [17]. These kinds of features make syngeneic and xenograft mouse models widely employed in pre-clinical approaches of new treatment protocols and adequate to unveil molecular traits and pathological aspects similar to HCC patients. However, it is still uncertain whether morphologic, genomic, and molecular aspects of engrafted HCC tumors remain similar to samples obtained from patients [16].

To establish a reliable orthotopic PDX model, an early study by Sun et al. [22] evidenced that surgically removed HCC samples, further implanted into BALB/c nude mice and selected according to its invasive potential, resemble translational features regarding morphological aspects and increased alpha-fetoprotein expression (Table 1). Besides, the LCI-D20 model showed take rates of 100% and transplantability through mouse generations, as well as spontaneous liver, lung, and lymph nodes metastasis after 6–24 weeks of protocol. Regarding the metastatic potential, Genda et al. [23] yielded a PDX model with an orthotopic injection of Li7 and KYN-2 cells into *scid*-mutated mice and showed their metastatic potential, with 50% of the engrafted animals showing intrahepatic micrometastasis after 6 weeks. Besides, in vivo and in vitro assays unravel an underlying p160ROCK-dependent mechanism in the metastatic activity of Li7 cells by suppressing Rho signaling (Table 1) [23]. Likewise, the PDX mouse model with HCC samples obtained by needle biopsies provides a striking similarity to the original biopsies by upregulating molecular pathways related to hypoxia, cell cycle progression, and epithelial-to-mesenchymal transition, even after at least 6 retransplantations into NOD-*scid* mice (Table 1) [24]. A NOD-*scid* mouse model displaying an impairment in the interleukin 2 receptor tends to increase HCC engrafted, making it a reliable model to evaluate the tumoral behavior alterations in a human immune microenvironment [25]. Indeed, the tumoral microenvironment associated with the immune background enhances tumoral growth, suggesting that HCC exerts a survival strategy of modulating immune checkpoints and attenuating cytotoxic T cell activity. Hence, the plasma levels of pro-inflammatory cytokines, such as tumor necrosis factor-α and human interferon-γ, increase (0–4 weeks of protocol) followed by a marked decrease (4–8 weeks of protocol), mimicking the HCC survival behavior in HCC patients [24]. Although these models do not resemble the whole landscape of tumoral-immune dynamics, HCC establishment requires a short experimental time (compared to chemical and diet-induced models, for example), maintaining key features of the derived tumor. In this scenario, the PDX mouse models represent a promising and translational strategy for discovering new drug therapies and the pivotal molecular mechanisms underlying the HCC development since this model resembles some of the genomic, morphological, immunological, and microenvironmental tumor characteristics observed in patients.

### 2.2. Chemical-Induced Rodent Models

#### 2.2.1. Diethylnitrosamine (DEN)

Diethylnitrosamine (DEN or DENA, PubChem CID:5921), which is also known as N-nitrosodiethylamine (NDEA), is the most prominent and widely applied xenobiotic in chemically induced models of HCC. Although the daily human ingestion of total N-nitrosamines usually occurs in low microgram (µg) ranges, reaching 0.5 to 1.0 µg/day, DEN holds the “Group 2A: probably carcinogenic to humans” classification according to the International Agency for Research on Cancer [28,29,30]. Both volatile and non-volatile nitrosamines account for human exposure, mostly through oral and respiratory routes, as they can be detected (>0.1 µg/kg) in tobacco smoke, food additives, and cured or smoked meat products as either naturally occurring compounds or formed after food processing [29,30,31,32]. There is plenty of in vivo evidence showing that DEN bio-activation occurs primarily in the liver (by the hepatocytes), mostly mediated by cytochrome P450 (CYP) 2E1. Thus, the constitutive activity of this cytochrome is strongly correlated to DEN-related outcomes on tumorigenesis as incidence and severity (number and size) in rodents [33,34]. DEN undergoes alpha-hydroxylation and dealkylation reactions, thereby producing the unstable ethyl diazonium hydroxide molecule that may generate highly reactive carbonium ions, oxygen (ROS) and nitrogen (RNS) species [35]. These highly reactive metabolites may bind to different biomolecules, including DNA and proteins. DNA alkylation or oxidation induced by DEN—such as the formation of O^6^-ethylguanine and O^4^- and O^2^-ethylthymine adducts mainly in centrilobular (zone 3) hepatocytes - may contribute to genomic instability, DNA damage, mutation, and tumor initiation [35,36,37,38]. Oxidative damage in proteins, such as conversion of protein thiol (-SH) groups to disulfides, is also featured in the liver after DEN exposure and may have direct implications on protein function and cell signaling [39]. As DEN was found to be a complete carcinogen in classical rodent bioassays (*i.e*., a chemical that can induce HCC development without the association of secondary chemical or surgical procedure as a promoter), this N-nitrosamine was widely applied as an “initiating agent” in the past few years within a myriad of protocols in mice and rats (Table 2). This chemical literally “initiates” the hepatocarcinogenic process by the production of a stable, heritable mutational change in the target cell (hepatocytes). Although it is not yet clear if this genomic alteration activates or inactivates one (or more than one) oncogene or tumor suppressor gene at the level of a single hepatocyte, it is mostly accepted that chemically induced preneoplastic lesions and HCC itself may clonally expand from this single DEN- “initiated” hepatocyte [40]. Nonetheless, when administered in drinking water or single or few non-necrogenic intraperitoneal (i.p.) injections to juvenile/adult mice, a long latency time is necessary to achieve a high burden of neoplastic lesions (Table 2).

Mindful of these findings, the studies of Vesselinovitch et al. shed light on the kinetics of using neonatal mice instead of juvenile/adult rodents. The main advantage of using neonatal model protocols in mice, also known as the “infant model”, is the hepatic postnatal development context [42,60]. Compared to the adult liver, hepatocyte proliferation rates are higher in the liver of neonatal mice [61]. Thus, when given at low doses ranging from 1 to 50 mg/kg body weight to neonatal mice at 15–20 postnatal days, the pro-proliferative hepatic context promotes the clonal expansion of DEN-initiated hepatocytes, ultimately favoring hepatocellular (pre)neoplastic lesion development and shortening the time for HCC emergence compared to juvenile/adult animals. According to the findings of Vesselinovitch et al. [42], mice display a progressively lower HCC incidence as the age at DEN administration increased from neonatal (46–69%) to juvenile/adult mice (9–10%) in a strain- and dose-dependent manner. However, the fact that the latency time for HCC development following neonatal DEN administration remains long inspired the use of different types of promoters (i.e., substances or procedures that enhance tumorigenicity when administered after a carcinogen) and the establishment of multi-stage protocols. The features of these combined chemical and/or surgical procedures will be discussed in Section 2.2.2, Section 2.2.3 , Section 2.2.4 and Section 2.2.5. In multiple weekly administrations in mice and rats (Table 2), DEN also acts as a hepatotoxicant by causing damage and necrosis. These cellular processes trigger a progressive inflammatory response that may lead to extracellular matrix (ECM) accumulation, leading to fibrosis or cirrhosis (protocol-dependent) [49,52]. The chronic pro-inflammatory context, resulting in elevated levels of hepatomitogen cytokines, may promote clonal expansion of DEN-initiated hepatocytes by paracrine signaling [62], increasing the burden of neoplastic lesions in a shorter time (100% of animals at 20–24 weeks post-initiation) (Table 2), an effect similarly obtained by using 2-stage protocols with fibrogenic promoters, such as carbon tetrachloride (CCl_4_) and thioacetamide (TAA) [49,52,53].

**Figure 1:**
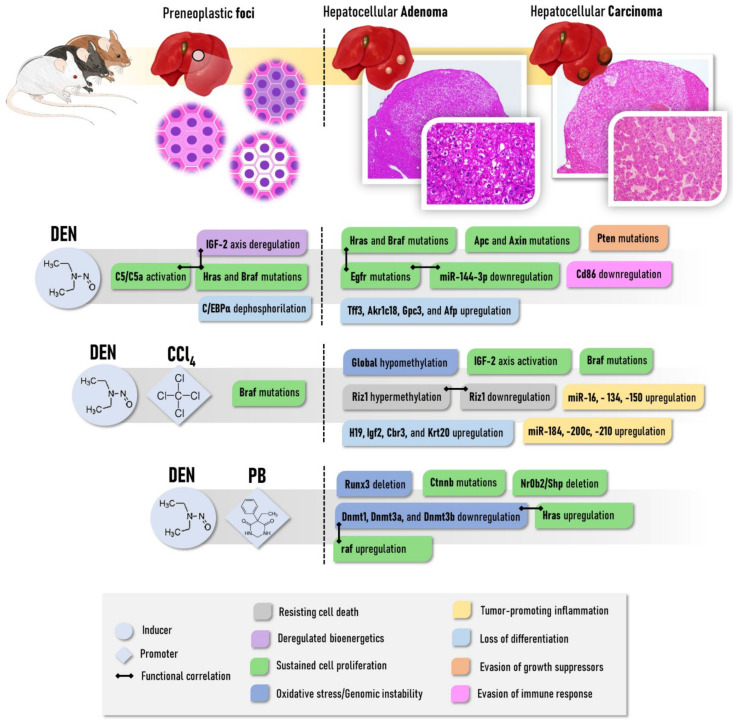
General depiction of the main molecular alterations and functional hallmarks involved in the development of preneoplastic (foci) and neoplastic (adenomas and carcinomas) lesions in widely-applied chemically induced models in mice. Strain-related and protocol-related variations should be considered. At late stages, molecular alterations are usually screened in a pool of neoplastic alterations, not considering if they are benign or malignant. CCl_4_: carbon tetrachloride; DEN: diethylnitrosamine; PB: phenobarbital. The figure was composed with the aid of illustrations from the SMART-servier Medical Art available at https://smart.servier.com/ (accessed on 15 January 2021).

Concerning the early molecular alterations caused by non-fibrogenic and subnecrogenic DEN administration in the liver, Watanabe et al. [63] revealed some biologically relevant mRNA networks both 4h and 28 days post-initiation in mice. Most of these genes showed a dose-dependent increase after 4 h, but not after 28 days. At both time points, genes were associated with cancer (i.e., *Fos*, *Jun*, and *Myc* oncogenes), cell cycle arrest, and cell death (i.e., *Bax*, *Cdkn1a*, *CCng1*, and *Gadd45*) gene expression. Sequentially, the first and smallest morphologically recognizable lesion in chemically induced models of hepatocarcinogenesis in rodents are the preneoplastic foci, also called altered hepatocyte foci, AHF. In general, foci present clear phenotypical variations and are usually classified as basophilic, eosinophilic, or clear cell foci according to the tinctorial characteristic of most hepatocytes in Hematoxylin and Eosin (HE)-stained sections [55]. These phenotypes seem not to occur at random, considering that the cell lineages that originate from these lesions are theorized to undergo a “metabolic turnover”. At first, DEN increases insulin growth factor 2 (IGF-2) levels, and IGF-2 downstream signaling decreases glucose-6-phosphatase (G6Pase) activity, promoting the emergence of glycogen storage phenotypes (eosinophilic and clear cell). The strong eosinophilia may result from the enhanced smooth endoplasmic reticulum (ER), peroxisome, or mitochondria. IGF signaling also promotes the Ras/Raf mitogen-activated signaling cascade, enhancing cell proliferation. Progressively, foci shift from anabolic to catabolic glucose metabolism to fuel cell proliferation, giving rise to the basophilic phenotype [64,65,66]. Along with the deregulated energetics hallmark, some AHF display *Hras* (10% of G6Pase-negative foci) and *Braf* (80–90%) oncogene mutations, which may provide a proliferative and growth advantage to these foci as late-stage neoplastic lesions also display these molecular alterations in higher frequency [67,68] (Figure 1). In this respect, *Braf* mutations are proposed to induce ERK1/Akt hyperphosphorylation and the induction of pro-survival/pro-proliferative complement component C5/C5a in basophilic foci [68] (Figure 1). For these reasons, AHF are generally considered putative preneoplastic lesions in chemically induced models, although the importance of morphologically similar lesions (glycogen-storing foci and small-cell change) is not completely understood in human hepatocarcinogenesis [65]. The molecular events that explain the stepwise progression of AHF to HCC are not fully unveiled, but recent findings indicate that some hepatocytes of DEN-induced AHF presenting oncogenic dephosphorylation of CCAAT/enhancer-binding protein alpha (C/EBPα) acquire a “stemness” feature, being classified as potential tumor-initiating hepatocyte (PTIH) [69]. Similar events were also described in the early and late stages of aggressive human HCC, suggesting that the preneoplastic foci with PTIHs are the origin of mouse HCC [69] (Figure 1).

In medium-term post-initiation timepoints (22–24 weeks), DEN has minimal effect on global miRNA expression and methylation profile in the liver, as only 8 miRNAs were upregulated and global/gene-specific methylation remained unaltered [70,71] (Figure 1). In more advanced stages, in a recent genome-wide investigation of stochastic point mutations, a high burden of potential coding alterations was observed in tumors (benign and malignant) harvested at 24–40 weeks post DEN initiation in C3H mice [38]. More than 80% of DEN-induced tumors had an activating hotspot mutation in either *Hras* or *Braf*, and around 20% of samples carried an activating mutation in *Egfr*. In addition, truncating mutations of gene suppressor *Apc* were exclusive to HCCs (21%). These alterations were considered putative oncogenic drivers of HCC in the DEN-induced model, as they may lead to the constitutive activation of Ras/Raf/MEK/ERK and Wnt/β-catenin signaling pathways, deregulating cell proliferation, growth, and survival processes. The downregulation of tumor suppressor miR-144–3p, as observed in human HCC, may also be accounted for Ras/Raf/MEK/ERK pathway activation in DEN-induced HCC as this miRNA downregulates *Egfr* [72] (Figure 1).

It is noteworthy that, as the occurrence of some mutations increased from benign to malignant tumors, also considering that some HCCs had a “nodule-in-nodule” morphological appearance at late stages, it has been hypothesized that the stepwise progression from benign tumors to HCC, similarly to the corresponding human disease. Another mutational profile also addressed activating *Braf* mutations in 89% of DEN-induced tumors carrying the V637E substitution, equivalent to the human V600E BRAF mutation. Of note, activating mutations in *Pik3ca* and inactivating mutations in the tumor suppressor *Pten* (PIK3CA inhibitor), involved in cell growth and angiogenesis, were also observed in 16% of tumors. Although common in the corresponding human disease, *Tp53, Tert*, and *Ctnnb1* mutations were not observed. Concerning the Wnt/β-catenin pathway, 5% of tumors displayed inactivating mutations in the tumor suppressor *Axin1* gene [73]. The transcriptomic profile of DEN-induced tumors sampled 44 weeks post-initiation (benign/malignant) also evidenced differential expressions of fetal/neonatal genes, such as *Tff3*, *Akr1c18*, *Gpc3*, *Afp*, and *Abcd2*, which are involved in robust physiological proliferative responses of undifferentiated cells [74]. Compared to the other 9 genetically engineered mouse models, DEN-induced tumors in mice showed markedly lower expression of *Cd86*, which is an immune-checkpoint stimulator, a feature predictive of poor prognosis concerning immunotherapy strategies [75] (Figure 1). Of note, comparative analysis revealed that the transcriptomic profile of DEN-induced HCC is similar to the poorer survival group of human HCCs [76].

Ultimately, regarding the molecular alterations observed in models of multiple necrogenic DEN administrations, Liu et al. [77] characterized gene expression profiles in rats during the progression from liver cirrhosis to malignant lesions, also comprising adenomas, early and late HCCs. Compared to the cirrhotic stage, transcriptomic changes in late HCCs were increased by 32–46%, as 999 and 906 mRNAs were up- and downregulated, respectively. Interestingly, all stages shared 349 upregulated and 345 downregulated genes, which were mainly associated with fat metabolism (*Scd2*, *Fap4*, and *Fabp5*, upregulated), oxidative stress (*Akr1b7*, *Akr1b8*, and *Aldh3a1*, upregulated), anti-oxidant defense (many members of glutathione axis, as *Gstm3*), ECM synthesis (*Itga6*, *Lamc1*, *Col1a1*, and *Spp1*), cell growth, proliferation and migration (upregulation of many annexin isoform-coding genes, such as *Anxa1*, *Anxa2*, *Anxa3*, *Anxa5*, and *Anxa7*).

As further presented (Section 2.2.2, Section 2.2.3, Section 2.2.4 , Section 2.2.5, Section 2.2.6, Section 2.2.7 and Section 2.2.8), there is a myriad of protocols applied in chemical-induced models (e.g., different chemical compounds, doses, frequencies of administration, etc.), and rat/mouse strains used (less or more susceptible), resulting in a clear methodological heterogeneity, and in the absence of a standard model. In general, regardless of the chemically induced protocol chosen, the models depicted in Section 2.2, mainly those induced by DEN, have been widely applied for the screening of predisposing and chemopreventive agents [57,78,79,80,81,82,83].

#### 2.2.2. Carbon Tetrachloride (CCl_4_)

In chemically induced rodent models, another widely applied xenobiotic is CCl_4_ (PubChem CID:5943). This haloalkane, which is usually administered in multiple intragastrical or intraperitoneal doses, is considered a promoter in 2-stage hepatocarcinogenesis models after DEN initiation (Table 2). CCl_4_ is metabolized in the hepatocytes by CYP2E1 to form the highly reactive oxygen trichloromethyl (*CCl_3_) and trichloromethyl peroxyl (*OOCCl_3_) radicals that promote lipid/protein damage and hepatocyte death, triggering an inflammatory response [84]. Oxidative stress, cell death, and inflammatory mediators are the stimuli for hepatic stellate cell (HSC) activation and collagen synthesis, ultimately leading to liver fibrosis and cirrhosis (a scenario that is absent in models using single or some DEN administrations) [5,85]. The establishment of a CCl_4_-induced pro-inflammatory and pro-fibrogenic background is thought to promote the clonal expansion of DEN-initiated hepatocytes, increasing the incidence of adenomas and HCC by 87.5% and 50%, respectively, compared to mice receiving only DEN at 22 weeks post-initiation [48]. A similar increase in neoplastic lesion burden is also observed in rats, suggesting a CCl_4_-mediated acceleration of HCC development [56,86] (Table 2).

Although *Braf* mutations are dependent on the genotoxic mechanism of DEN, which is absent in the CCl_4_ regimen, Yamamoto et al. [68] showed that these alterations are maintained in (pre)neoplastic lesions induced by the DEN/CCl_4_ protocol, suggesting the potential importance of this oncogene in tumors arising in a fibrotic context as well (Figure 1). Different from models using initiating non-fibrogenic DEN protocols, epigenetic alterations are key events in DEN/CCl_4_-induced models of fibrosis-associated hepatocarcinogenesis. In this scenario, adenomas and carcinomas feature global DNA hypomethylation and decreased histone 3 lysine 9 trimethylation (H3K9me3), which are indicators of genomic instability. Furthermore, HCCs present promoter hypermethylation and functional downregulation of tumor suppressor *Riz1*, which was associated with accelerated tumor burden. Some of these alterations were also observed in the fibrotic tissue surrounding the lesions while absent in non-fibrotic tissue in DEN-initiated animals [70] (Figure 1). In medium-term post-initiation timepoints (22 weeks), the DEN/CCl_4_ protocol led to a distinct profile of 25 upregulated oncogenic and pro-fibrotic miRNAs, which are associated with proliferation, apoptosis, inflammation, and fibrosis functional networks, and thus also correlated with the increased neoplastic lesion burden [71] (Figure 1). Tumors arising from the CCl_4_-induced fibrotic background also showed deregulated expression of oncofetal genes, such as the upregulation of *H19*, *Igf2*, *Cbr3*, and *Krt20* compared to DEN-induced tumors (Figure 1). In particular, continuous activation of the IGF-2-mediated axis in both tumors and surrounding fibrotic parenchyma, which is only observed in the early stages of mice submitted to the DEN protocol, mediates excessive hepatocyte proliferative stimuli following CCl_4_-induced chronic liver injury, which could contribute to the increased (pre)neoplastic lesion burden [66,74]. Even though CCl_4_ is routinely applied as a promoter by establishing necrogenic, inflammatory, and fibrotic responses, some protocols use this haloalkane as a complete carcinogen, as some hepatocytes are initiated by adduct formation between *CCl_3_ radical and DNA while presenting decreased neoplastic lesion burden compared to DEN and DEN/CCl_4_ protocols (Table 2) [48,74,84].

#### 2.2.3. Thioacetamide (TAA)

TAA (PubChem CID 2723949) multiple i.p. injections or medium-long term administration in drinking water mimics chronic liver damage, fueling the development of DEN-induced (pre)neoplastic liver lesions in an inflammatory scenario in rodents (Table 2). TAA undergoes metabolic activation by CYP2E1 in the liver, generating S-oxide (TASO) and S, S-dioxide (TASO(2)) reactive compounds that sequentially exert amine lipids, protein damage, cell death, inflammatory response, HSC activation, excessive ECM synthesis, and fibrosis/cirrhosis in a protocol-dependent manner [87]. Most of the relevant histopathological and mechanistic data on DEN/TAA-induced hepatocarcinogenesis is derived from rat models. In short- and medium-term experiments, the screening of glutathione-S-transferase pi (GST-P)-positive foci by immunohistochemistry, which is not detected in normal liver, is widely applied and well-accepted in rat models. Placental GST-P is a long applied and accurate marker for the identification of putative preneoplastic lesions, as classical findings demonstrated that known hepatocarcinogens and hepatopromoters enhance the induction of GST-P+ foci, while non-hepatocarcinogens and non-hepatopromoters do not. In addition, late-stage neoplastic lesions, as liver adenomas and carcinomas, feature increased GST-P expression as well [88,89]. Noteworthy, TAA administration after single DEN administration increased the number and liver area occupied by GST-P+ foci by 5- and 10-fold compared to animals that were only initiated by DEN [90].

**Figure 2:**
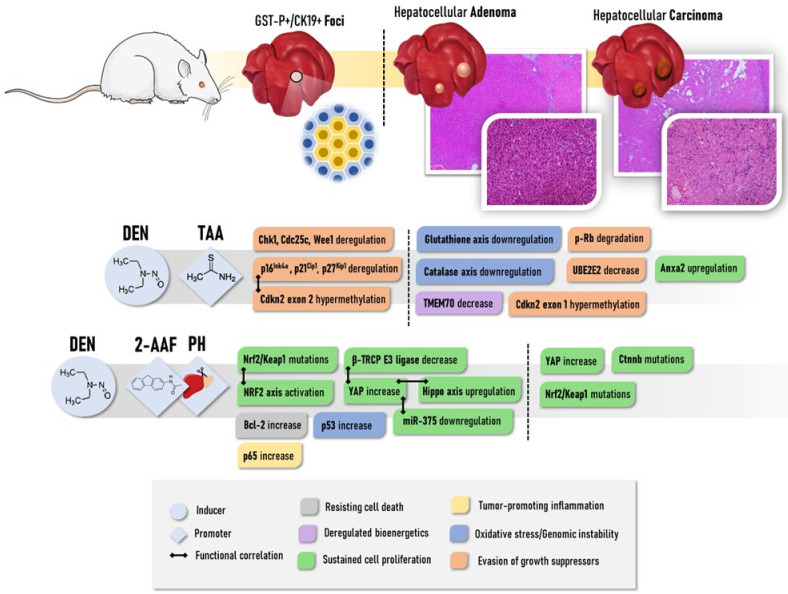
General depiction of the main molecular alterations and functional hallmarks involved in the development of preneoplastic (foci) and neoplastic (adenomas and carcinomas) lesions in widely-applied chemically induced models in rats. Strain-related and protocol-related variations should be considered. At late stages, molecular alterations are usually screened in a pool of neoplastic alterations, not considering if they are benign or malignant. 2-AAF: 2-acetylaminefluorene; DEN: diethylnitrosamine; PH: partial hepatectomy; TAA: thioacetamide. The figure was composed with the aid of illustrations from the SMART-servier Medical Art available at https://smart.servier.com/ (accessed on 15 January 2021).

In the early stages of hepatocarcinogenesis, TAA promotion deregulated the expression of many G1/S and G2/M proteins, of which expression either increased or decreased, contributing to the clonal expansion of hepatocytes populations featuring checkpoint disruption and genomic instability in GST-P+ foci (Figure 2). Epigenetic alterations may be involved in these TAA-induced promoting mechanisms, such as the exon 2 of Cdkn2a featured hypermethylation, which was not found in animals submitted only to DEN initiation [90,91]. These early cell cycle alterations in GST-P+ foci may contribute to neoplastic lesion emergence since 50% of DEN/TAA-induced poorly differentiated HCCs display hypermethylation of exon 1 of *Cdkn2a (*Figure 2). The degradation (hyperphosphorylation) of tumor suppressor Retinoblastoma protein (pRb) (Figure 2), which has a pivotal role in the negative control of the cell cycle, is progressively increased in DEN/TAA-induced liver adenomas and carcinomas, while absent in early fibrotic stages [58]. More recently, Mizukami et al. [92] showed that TAA promotion might decrease the expression of TMEM70 and UBE2E2, involved in oxidative phosphorylation and cell cycling, in GST-P+ lesions by hypermethylation (Figure 2). Findings indicated that these alterations were acquired in early preneoplastic (foci) and increased in late neoplastic stages (adenomas and carcinomas) [92].

Deregulation of the antioxidant axis, leading to increased oxidative stress, is also proposed to have key roles during DEN/TAA-induced hepatocarcinogenesis in rats [57]. Repeated administration of TAA depletes different anti-oxidant systems, decreasing total glutathione content and mRNA/activity of catalase, glutathione-S-transferase, and glutathione peroxidase [57,93]. Interestingly, the upregulation of Anxa2 was also seen in the liver of DEN/TAA-induced cirrhosis/hepatocarcinogenesis at 26 weeks post-initiation [57] (Figure 2). A hierarchical cluster analysis revealed that neoplastic lesions arising from both CCl_4_-induced and TAA-induced cirrhotic backgrounds in mice had similar mRNA expression profiles, sharing the selective activation of the IGF-2 pathway in comparison to the tumors that emerged from the non-cirrhotic scenario [74]. Like CCl_4_, repeated treatment with TAA is proposed to have initiating potential, while not as pronounced as DEN, as many DNA damage-inducible genes are upregulated, and (pre)neoplastic lesions (protocol-dependent) are observed in response to different TAA regimens in both rats and mice [74,94].

#### 2.2.4. Phenobarbital (PB)

Phenobarbital (PubChem CID 4763) has been used for several decades as a promoter of 2-stage hepatocarcinogenesis models in rats and mice [41,45,50,95,96,97]. This non-genotoxic barbiturate is usually given in low doses in drinking water or diet after the administration of an initiating carcinogen like DEN (Table 2). In contrast to multiple DEN administration and CCl_4_ or TAA regimens, PB promotion leads to a non-fibrogenic hepatic event. While the mechanisms regarding PB-related promotion are not fully elucidated, the hepatic context of PB administration positively selects hepatocytes harboring the activating mutations of the *Ctnnb1* gene, which lead to activation of Wnt/β-catenin signaling in about 80% of neoplastic lesions [98] (Figure 1). Moreover, most preneoplastic foci and neoplastic lesions induced by DEN/PB protocol present strong eosinophilia, whereas the common DEN-induced basophilic AHF is not as frequent [99]. For this reason, some authors denominate PB as a “tumor selector” or “selective promoter” rather than a classical promoter [100], considering that *Hras* mutations, which are frequent in DEN-induced tumors, are infrequent in DEN/PB-induced tumors. Moreover, *Ctnnb1* mutations are absent in protocols using only DEN as tumor initiator [38,100].

PB administration induces CYP450 enzymes, increasing the metabolic capacity of hepatocytes, which could increase the bio-activation of hepatotoxic drugs, thus enhancing their genotoxic/cytotoxic effects [101]. Some 2-stage rat protocols apply 1-week-long 0.05% PB interventions after DEN initiation and preceding promoter administrations, such as TAA (Table 2) [58]. It is suggested that the activation of the nuclear constitutive active/androstane receptor (CAR), which is involved in the induction of CYP450 enzymes, is essential for liver tumor promotion by PB in mice since CAR knockout (KO) mice led to the absence of (pre)neoplastic lesions in DEN/PB-induced protocol [102]. Furthermore, the sex-dependent interplay between CAR and β-catenin, being pronounced in male mice, may regulate enzyme induction and hepatocyte proliferation [103], which could explain the outgrowth of HCC with predominant eosinophilic phenotype and activated β-catenin signaling. More recently, Aleksic et al. [37] found that chromosomal instability may precede the outgrowth of *Ctnnb*-mutated hepatocytes. At early tumorigenesis stages, 29% of neoplastic alterations had chromosomal gains and/or losses, which increased in late stages, as 92% of tumors harbored these alterations. Among those, the loss of distal chromosome 4q, including the tumor suppressors Runx3 and Nr0b2/Shp, was an early and persistent event during DEN/PB-induced hepatocarcinogenesis (Figure 1). In contrast, Ctnnb occurred at high frequency only at late stages. In addition, PB and other chemicals have been shown to block gap junctional intercellular communication (GJIC) to exert their promotional activity. Although all mechanisms are not fully understood, Moennikes et al. [104] demonstrated that functional connexin 32 (Cx32) is required for tumor promotion by PB, considering that Cx32 null mice did not feature marked increases in size, volume, and/or the number of (pre)neoplastic lesions in response to PB promotion compared to Cx32 wild-type mice. In contrast, Cx26 KO mice have only minor effects on DEN/PB-induced mouse hepatocarcinogenesis [105].

Regarding the protocols (Table 2), PB administration after different DEN initiation protocols leads to 3–5-fold and 4–6-fold increases in the number of GST-P+ preneoplastic foci and HCCs in different rat strains in a concentration-dependent and time-dependent manner, respectively, compared to DEN counterparts [50,97]. The PB promotion effects in mice depend on the timing of DEN initiation. When given to mice submitted to DEN initiation at 2 weeks of age, PB did not alter (C3H/HeJ) or paradoxically attenuated tumorigenesis some mice strains, including C57BL/6J and B6C3F1, a crossbreed of C3H/HeJ and C57BL/6, whereas promoted in other strains, in particular, BALB/c and CD1 [45,47,95,106,107]. While apparently strain-dependent and not deeply investigated, some authors hypothesized a “feminizing” effect of early PB administration [95,106], also considering the key effects of sex hormones on hepatocarcinogenesis (see Section 2.2.7). Nonetheless, when 4–6 weeks-old mice are initiated with DEN and subsequently submitted to PB exposure (Table 2), the incidence of adenomas and carcinomas increases by 50–90% and 60–100% in a time-dependent and strain-dependent manner compared to animals that only received DEN, indicating marked tumorigenesis promotion [41]. DBA/2, C3H/He, and BALB/c mice showed increased sensibility to PB promotion, while C57BL/6 mice were rather refractory [41,108]. The results were partly attributed to the potential inter-strain differences on (1) PB metabolism, as PB serum levels were increased in DBA/2 and compared to C57BL/6 [41], and (2) PB-induced deregulation of the methylation status of key driver genes, as B6C3F1 is less capable of maintaining methylation balance compared to the C57BL/6 strain [109]. DNA methyltransferase genes (*Dnmt1*, *Dnmt3a*, and *Dnmt3b*) are downregulated in B6C3F1 mice [110] (Figure 1). The enzymes coded by these genes possess CAR response elements (CAREs), reinforcing PB as a CAR agonist. The multiple subsequent genomic events resulting from the deregulation of methylation status may be involved in tumorigenesis in this strain, such as hypomethylated *Hras* and raf upregulation (Figure 1) and alterations other genes involved in cell cycle, apoptosis, angiogenesis, invasion/metastasis [109,110,111]. The several hepatocarcinogenesis-related susceptibility/resistance loci mapped in these strains may also contribute to the aforementioned differences in response to PB promotion [112] (see Section 2.2.8)

#### 2.2.5. Resistant Hepatocyte Model

One of the most applied models for the study of multistage chemical hepatocarcinogenesis is the Solt-Farber model in rats, which is also known as the “resistant hepatocyte (RH) model” [113,114]. In general, the RH model relies on a chemically induced genotoxic insult as an initiator followed by a regenerative response under a chemically induced selective pressure [115]. While several other chemicals were employed in the 1980s [114,115], the initiation protocol is usually accomplished by a single DEN dose followed by a short-term intragastrical or dietary administration of 2-acetylaminofluorene (2-AAF, PubChem CID: 5897) [113,116]. Under the 2-AAF regimen, rats are subsequently submitted to 70% partial hepatectomy (PH), which was introduced by Higgins and Anderson [117] to induce liver regeneration. 2-AAF administration exerts a mito-inhibitory selective property, thus blocking the proliferation of non-initiated hepatocytes and stimulating the DEN-initiated cells that are “resistant” to 2-AAF toxicity. Under the influence of the PH-induced proliferative stimulus, the selective expansion of these initiated hepatocytes results in preneoplastic foci and hyperplastic nodules, some of which may progress into HCC [113,114]. The model was first established in the susceptible Fisher-344 rat strain and later adapted to other rat strains, including the intermediate susceptible Wistar strain [118]. As the main outcomes of this protocol (Table 2), enzyme-altered preneoplastic lesions featuring an elevated expression of gamma-glutamyltranspeptidase (y-GT) and GST-P, visible primary HCCs and few metastatic tumors are observed in short- or medium-term studies [114,116,119]. About 95–98% of these enzyme-altered foci/nodules are proposed to suffer spontaneous remodeling to normal-appearing hepatocytes, called “remodeling lesions”. On the other hand, only a small portion may progress to HCC, denominated as “persistent lesions”. These lesions display differences regarding key molecular pathways that could direct their progression. Persistent GST-P-positive lesions have increased proliferative indexes, p53 accumulation, increased anti-apoptotic Bcl-2 staining, and enhanced p65 immunostaining compared to the remodeling ones, which showed increased apoptotic indexes [120] (Figure 2).

Moreover, the stem/progenitor cell origin of HCC has been proposed in this model [121,122]. In rodents, so-called oval cells, small periportal ductular-like progenitor cells that give rise to hepatocyte and bile ductular cell populations, are often observed during the early hepatocarcinogenesis stages in the RH model. The oval cells have been suggested to present natural resistance to mito-inhibitory chemicals and may originate hepatic tumors under the regenerative stimulus. Additionally, HCCs that arise in the RH rat model have shown similar immune–expression of oval cells markers, such as keratin (K)7, K19, and Ov6, indicating its possible progenitor cell derivation [121,122,123]. Perra et al. [124] have shown the involvement of the Hippo signaling pathway member YAP during the early stages of hepatocarcinogenesis in the RH model. This key transcriptional co-activator was found to be overexpressed at the translational level in both early and late hepatocarcinogenesis stages (Figure 2). In parallel, YAP target genes were also upregulated in preneoplastic foci and in oval cells. Moreover, the experimental disruption of YAP-related transcriptional complexes significantly reduced preneoplastic foci development and oval cell proliferation in rats, indicating the involvement of YAP in liver tumorigenesis. The overexpression of YAP in the early stages was associated with the downregulation of the β-TRCP E3 ligase and miR-375, known to negatively regulate this protein [124] (Figure 2). Of note, enhanced YAP expression was also featured in early human dysplastic nodules and adenomas [124] (Figure 2). Petrelli et al. [125] investigated the involvement of miRNA-gene interactions during the early stages of HR-induced liver carcinogenesis. Noteworthy, 80–85% of the most upregulated/downregulated genes in rat HCC were already altered in early K19-positive preneoplastic nodules. Among the deregulated networks, the activation of the nuclear factor erythroid-related factor 2 (NRF2) pathway and upregulation of the miR-200 family were described in K19-positive nodules. Reinforcing the translational value of the RH model, 78% and 57% of differentially expressed genes and miRNAs in rat HCC have been previously associated with human HCC, respectively. NRF2 pathway upregulation is indeed involved in early Nrf2/Keap1 mutations, which are observed in 71% of early preneoplastic lesions, in 59.3–78.6% of HCCs, and in 50% of lung metastases of HCC-bearing rats (Figure 2). Although the role of NRF2 as a tumor suppressor or oncogene is still controversial, data suggest an oncogenic role of this transcription factor as it may contribute to the clonal expansion of preneoplastic hepatocytes to HCC. Unlike human hepatocarcinogenesis, β-catenin gene mutations do not occur in the early stages of the RH model, and only in 18.5% of HCCs [126] (Figure 2).

#### 2.2.6. Aflatoxin B1

Dietary intervention with low concentrations of aflatoxin B1 (AFB1, PubChem CID: 186907), its metabolites (aflatoxicol), or other aflatoxins (such as G1) has been extensively tested in rodent bioassays for hepatic carcinogenicity [127,128,129,130]. Although AFB1 is classified as a group 1 human carcinogen [28], and the consumption of improperly stored aflatoxin-contaminated food is widespread in the world, the identification of human aflatoxin-associated HCC cases is difficult, considering the unclear history of exposure [131]. One of the main molecular alterations caused in humans by AFB1 exposure is the point mutation (G to T) at codon 249 in the TP53 tumor suppressor gene [132]. However, site-specific mutations within the comparable codon in the Tp53 gene are not frequent in AFB1-induced liver (pre)neoplastic lesions in rats [108,133]. In rodents, the early AFB1-related hepatocarcinogenic mechanisms may be associated with increased lipid peroxidation and inflammation, and impaired anti-oxidant response that may contribute to cell injury, DNA damage, and preneoplastic foci growth [134,135]. In AFB1-induced HCC, the transcriptomic analysis revealed that AFB1 accounts for extensive deregulation in the expression of both protein-coding genes and long non-coding RNAs (lncRNAs). Some AFB1-deregulated lncRNAs clusters were associated with modification of apoptosis-, cell cycle-, response to DNA damage stimulus-, and Wnt receptor signaling pathway-related protein-coding genes. Apoptosis is proposed to contribute to AFB1-induced hepatic carcinogenesis since anti-apoptotic (*Bcl2*, *Mapk8*, and *Nfkb1*) and pro-apoptotic genes (*Casp1*, *Il4*, and *Mpo*) were upregulated in the HCC samples [136].

#### 2.2.7. Miscellaneous Chemicals

Many other chemicals, including benzo(a)pyrene (BaP, PubChem CID: 2336), N-methyl-N-nitrosourea (MNU, PubChem CID 114836), and 1,2-dimethylhydrazine (1,2-DMH, PubChem CID: 1322), have been applied in classical bio-assays as initiator chemicals for the induction of enzyme-altered foci and tumors. Considering that these substances are not as efficient in inducing hepatic preneoplastic and/or neoplastic lesions compared to DEN-only protocols, they are usually combined with a chemically induced and/or surgically-induced cell proliferative promoting stimulus [59,137,138]. In a classical colon carcinogenesis bioassay, 1,2-DMH administration in Wistar rats led to increased oxidative stress, impaired anti-oxidant defense, upregulation of pro-apoptotic genes, and the development of few GST-P-positive foci in the liver 24 weeks after the carcinogen regimen [139]. In addition to the role of this hydrazine as an initiator, 1,2-DMH administration was proposed to promote a DEN-initiated bioassay by inducing CYP2E1, enhancing DNA adduct formation in the liver, and increasing the number of GST-P-positive foci [140].

#### 2.2.8. Impact of Genetic Background and Sex

**Figure 3:**
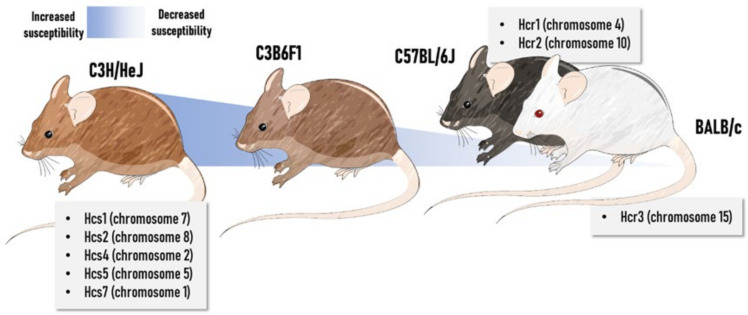
General depiction of some loci potentially involved in hepatocarcinogenesis susceptibility in widely-applied inbred and crossbred mouse strains. Hcs: Hepatocarcinogen susceptibility locus; Hcr: Hepatocarcinogen resistance locus. The figure was composed with the aid of illustrations from the SMART-servier Medical Art available at https://smart.servier.com/ (accessed on 15 January 2021).

There is a spectrum of paradigms involving mice-specific susceptibility to hepatocarcinogenesis models, not only including the chemically induced bioassays but also the genetically modified ones [112,141] since different mice strains serve as backgrounds for the latter. In this respect, intrinsic genetic factors may contribute to the previously mentioned responses to chemical initiators and promoters. Several quantitative trait loci of susceptibility (Hcs) or resistance (Hcr) have been mapped using recombinant congenic and inbred consomic strains. The greater liver cancer predisposition of the C3H/HeJ compared to C57BL/6J strain is mainly attributed to hepatocarcinogen sensitivity 7 (Hcs7) loci found in chromosome 1 [142]. The Hcs7^C3H^ allele was sufficient to confer susceptible traits to the C57BL/6 strain. Hcs7 may promote hepatocyte growth and proliferation in both normal and preneoplastic hepatocytes, apparently without affecting carcinogen metabolism and subsequent adduct formation [143,144]. Interestingly, Hcs7 encodes transcription factors, regulators of G-protein signaling, a member of the TNF ligand superfamily, and a receptor tyrosine kinase [142]. Other similar studies mapped many sensitivity loci in the C3H/He strain, whereas resistance loci were identified in both C57BL/6 and BALB/c strains, some of them carrying proto-oncogenes (such as c-jun and L-myc) [145,146,147,148,149]. In general, these genetic features may explain the fact that C3H/HeJ mice spontaneously develop HCC in a long-time latency, while incidence is low in crossbred C3B6F1 animals and extremely rare in C57BL/6 males [38]. The crossbred C3B6F1 strain, considered of intermediate susceptibility, is the default mouse strain for the National Toxicology Program. In rats, Hcs and Hcr loci were also identified in backcrosses and intercrosses experiments performed in susceptible F344 rats and resistant Brown Norway (BN) and Copenhagen (Cop) rats [150,151]. Moreover, in DHN strain, which is originated by inbreeding of Donryu colony, the Drh2 cluster located in rat chromosome 4 was closely associated by mapping analysis to suppression of (pre)neoplastic lesions during chemically induced hepatocarcinogenesis, controlling the expansion of GST-P positive foci and the emergence of HCC [152,153]. A general depiction of the main Hcs and Hcr loci in widely-applied mouse strains can be found in Figure 3.

One of the main advantages of using chemically induced models of hepatocarcinogenesis is the sex disparity feature reflecting the corresponding human disease. In men, both incidence and mortality rates for HCC are 2.8-fold higher compared to women [1]. In DEN-initiated models in mice, in particular when using CCl_4_ or PB as promoters, females develop HCC at a later age and with a lower incidence/multiplicity in comparison to males, in a strain-dependent, dose-dependent, and timepoint-dependent manner [42,55,62]. The roles of sex hormones on hepatocarcinogenesis are not fully understood in both human and animal models. It is reported that 17β-estradiol (E2) exerts an anti-inflammatory effect by inhibiting the nuclear transportation of the p65 subunit of NF-κB in macrophages (RAW 264.7), also reducing NF-κB-related DNA-responsive elements [154]. Heterotopic-engrafted ovariectomized/castrated BALB/c mice treated with E2 featured reduced volume of tumors by suppressing the alternative activation of tumor-associated macrophages into a pro-inflammatory profile in an IL-4-Jak1-Stat6-dependent mechanism [155]. In accordance with these findings, estrogen-related receptor-α (ERR-α) KO mice enhanced DEN-induced hepatocarcinogenesis in a neonatal mice model, increasing the incidence (100 and 25%, respectively) and multiplicity (~7 and ~2 tumor/liver, respectively) of tumors, in comparison to wild-type mice, attesting that ERR-α KO mice are susceptible to HCC initiation and progression. In addition, KO-ERR-α mice display increased nuclear recruitment of p65 subunit, increased level of DNA synthesis, and necrosis occurrence, suggesting a cytokine-driven compensatory proliferation mechanism that promotes hepatocarcinogenesis progression [156]. Thus, E2 is suggested to be one of the mechanisms responsible for the sex disparities observed in epidemiological and in vivo experimental model data, attenuating the HCC progression. Although the genetic basis of female resistance for hepatocarcinogenesis is not fully unveiled, the introgression of Hcs4 from BN rats in F344 background revealed that this locus of chromosome 16 may display resistance genes regulated by sex hormones. The gonadectomy of congenic F344.BN-Hcs4 rats during the establishment of resistant hepatocyte increased the development of (pre)neoplastic lesions in females while decreasing in males. In keeping with these findings, the administration of testosterone to gonadectomized F344.BN-Hcs4 females resulted in enhanced (pre)neoplastic lesion burden, similarly to parental F344 males, whereas the administration of E2 to gonadectomized F344.BN-Hcs4 males decreased (pre)neoplastic lesion emergence, relatable to parental BN females. These effects were accompanied by functional receptor modulation, in special ERR-α, indicating the potential modulation of sex hormone-sensitive gene (s) in this chromosome [151].

It is also suggested that androgens might be responsible for the sex disparities observed in humans and in vivo experimental models. The androgens exert their bioactive function by interacting with androgen receptors (AR), which then act as a transcription factor and induce the expression of key molecules associated with hepatocarcinogenesis [157]. However, it is still uncertain whether androgens and/or AR were responsible for inducing the HCC progression. Accordingly, it is observed that nuclear AR is overexpressed in ~33% of HCC samples when compared to noncancerous liver tissues (~2-fold), correlating to the poorer overall survival of patients and prognostic [158]. Additionally, it is observed that both male and female KO-AR (total or liver-specific) mice submitted to a DEN-induced hepatocarcinogenesis model featured similar serum testosterone levels, in addition to a longer latency period, with reduced incidence and size of tumors, when compared to male, female and littermates wild-type mice. This data suggest that AR rather than testosterone promotes HCC progression by modulating the oxidative-apoptotic axis [159]. Therefore, the sexual disparities observed in epidemiological and reflected in vivo experimental model is mainly related to the mechanisms of the E2 and AR by modulating the inflammatory-oxidant axis that turns the hepatic milieu susceptible to HCC emergence.

### 2.3. Diet-Induced Rodent Models

#### 2.3.1. NAFLD-Associated HCC Models

**Figure 4:**
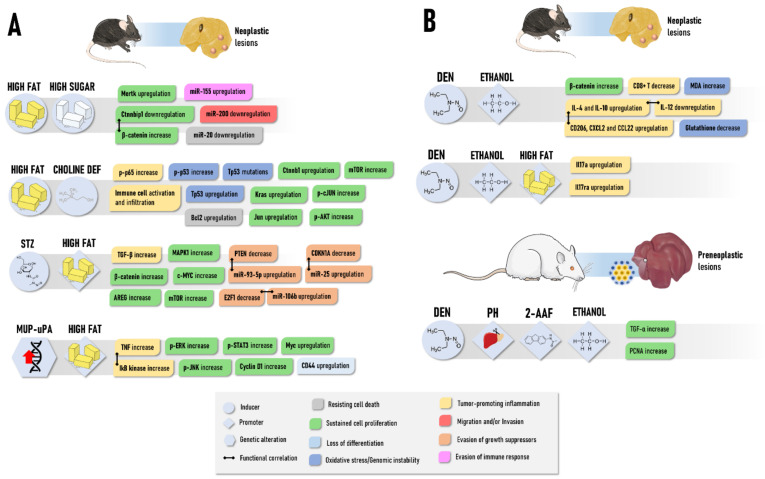
General depiction of the main molecular alterations and functional hallmarks involved in the development of neoplastic (adenomas and carcinomas) lesions in NASH-driven (**A**) or ALD-driven models (**B**) in mice and rats. NASH: Non-alcoholic steatohepatitis; ALD: Alcoholic liver disease. The figure was composed with the aid of illustrations from the SMART-servier Medical Art available at https://smart.servier.com/ (accessd on 15 January 2021).

In the last decade, a variety of suitable preclinical models mimicking NAFLD/NASH-driven HCC have been developed. As reviewed by Febbraio et al. [160], although none of the available models fully reproduce the broad range of complex events of NAFLD/NASH pathogenesis, presenting discrepancies in the presence/absence of obesity, insulin resistance, inflammation/ER stress, and NASH, most mechanistic data on NAFLD-associated hepatocarcinogenesis are derived from these mouse models (Figure 4A). In general, these bioassays are classified into (1) diet-induced, (2) chemically induced, and (3) genetically modified models, and (4) “hybrid” models combining these 3 interventions (Table 3). There is a great diversity of dietary ad libitum intervention models available in the literature, mostly displaying high sugar and/or fat contents. In general, diet-only interventions require a long period of latency, also presenting a highly variable tumor incidence, multiplicity, and size (Table 3). Despite this disadvantage, neoplastic lesions arise as part of the natural disease progression and do not require induction by a chemical carcinogen. In these models, the C57BL/6J strain is widely chosen because of its predisposition to developing insulin resistance and obesity [161]. More recently, Asgharpour et al. [162] showed that B6/129 mice, which are derived from a C57BL/6J and 129S1/SvImJ background, are more insulin-resistant, NASH-prone, and HCC-prone compared to their parental strains. In NASH-driven HCC in B6/129 mice, the transcriptomic analysis revealed the activation of both metabolic and oncogenic pathways, including nitrogen and amino acid metabolism, oxidative stress signaling, inflammation, cell adhesion, and ECM remodeling. Interestingly, tumors featured the upregulation of the proto-oncogene Mertk, which is a tyrosine kinase-coding gene involved in proliferation and invasion, and the downregulation of *Ctnnbip1*, a negative regulator of the β-catenin pathway. The comparison between the transcriptomic signatures of human HCC and NASH-driven HCC in B6/129 mice revealed close similarity to S1/2 subclasses of human HCC, which are characterized by WNT, MYC, and AKT pathway activation [162,163]. As demonstrated by Dowman et al. [164], some of these tumors showed nuclear accumulation of β-catenin protein, indicating Wnt pathway activation in mice as well. In the C57BL/6J strain, NASH-driven HCC featured miRNA deregulation, including the upregulation of miR-155, -193b, -27a, -31, -99b, -484, -574-3p, -125a-5p, and -182, and the downregulation of miR-20a, -200c, -93, -340-5p, and -720. Some of these miRNAs were proposed to have oncogenic or tumor suppressor activities, similar to the corresponding human disease [165] (Figure 4A).

Another commonly applied diet-induced bioassay for NAFLD/NASH-driven HCC modeling is the ad libitum feeding with the toxic choline-deficient high-fat (CDHF) diet [167]. The CD diet is known to exacerbate HF-induced NASH, as inadequate choline uptake impairs hepatic lipoprotein secretion and promotes oxidative damage caused by mitochondrial dysfunction and ER stress. Wolf et al. [167] demonstrated that the CDHF diet increased the incidence of HF-induced HCC by 10-fold (Table 3), unraveling an interaction between inflammatory cells (natural killer and CD8+ T lymphocytes) and hepatocytes that lead to liver damage, canonical NF-kB signaling activation hence promoting NASH-to-HCC transition (Figure 4A). Although CDHF-induced HCCs showed heterogeneous patterns of chromosomal aberrations, copy number changes revealed similarity with cryptogenic HCC in humans. These murine tumors also demonstrated deregulated protein and/or mRNA expression of many oncogenes, such as p-AKT, p-cJUN, p65, *Bcl2*, *Ctnnb1*, *Kras*, and *Tp53*, that also presented missense activating mutations. Interventions with L-amino acid-defined diets -in combination with the CDHF diet also fuel the HF-driven HCC burden by enhancing the NASH background [168].

In toxin-based approaches, the so-called Stelic Animal Model (STAM) of NASH-hepatocarcinogenesis is widely established [170,173,174] (Table 3). Low-dose administration of streptozotocin (STZ) in the first days of life of a mouse leads to oxidative injury in pancreatic islets and profound changes in hepatic transcriptomic profile [175,176]. This alkylating agent established diabetic conditions, usually absent in dietary interventions, which promote rapid lipogenesis, fatty acid oxidation, hepatocellular injury, and fibrosis [170,173]. In combination with the HF diet, STZ-administered mice display a higher and faster burden of tumors compared to HF diet-only interventions (Table 3), since mice display at least 4 detectable HCCs, and an average tumor growth rate of 150% from 16 to 20 weeks of age [170]. De Conti et al. [174] further characterized profound deregulation in miRNA-target networks in this model, including the upregulation of many miRNAs and the activation of major oncogenic pathways, including TGF-β, Wnt/β-catenin, ERK1/2, mTOR, and EGF signaling. In particular, E2F1, PTEN, and CDKN1A were directly targeted by the upregulation of miR-106b, miR-93-5p, and miR-25 in NASH-cirrhosis and full-fledged HCCs stages (Figure 4A). Interestingly, some of these upregulated miRNAs were also featured in human HCC, and progressive increase of their expression levels from the NAFLD/NASH (weeks 6–12) to HCC (week 20) stages, eliciting their importance during disease progression. Among the other toxins applied in association with dietary interventions (Table 3), CCl_4_ multiple administrations, in similar protocols as described in Table 2, are also chosen to increase not only the inflammatory/fibrotic context of NASH but also the neoplastic lesion burden [85,169].

Concerning NASH-related genetically modified models, the hepatocyte-specific *Pten* deficiency results in a fast induction of steatohepatitis, as hepatocytes acquire adipogenic-like features [171,177]. Given that *Pten* is also a tumor suppressor gene highly implicated in hepatocyte homeostasis, *Pten* null mice also have a high burden of hepatocellular neoplastic lesions (Table 3) [171]. More recently, Nakagawa et al. [172] developed a model combining HF diet feeding and MUP-urokinase plasminogen activator (uPA) transgenic mice. These animals have high levels of uPA, which induces transient ER stress and liver damage, that is also implicated in human NASH, leading to the development of indistinguishable NASH-related morphological and molecular hallmarks. Other authors also noticed a high burden of hepatocellular adenomas and carcinomas (Table 3), evidencing that uPA-induced ER-stress and HF have synergistic roles on both NASH development and HCC progression. These events were highly dependent on TNF production by inflammatory liver macrophages and TNF receptor 1 (TNFR1)-IkB kinase b (IKKb) signaling in hepatocytes.

In general, diet-induced protocols (Section 2.3) usually rely on the administration of “real-world” methodological approaches (fat, sugar and alcohol intake, as the corresponding human habits) as their main advantage. Moreover, key transcriptomic resemblances to human HCC are also observed [161,162]. Nonetheless, one should consider the long latency time to HCC emergence and highly variable incidence as the main disadvantages of these bioassays.

#### 2.3.2. ALD-Associated HCC Models

According to IARC, there is sufficient evidence in both humans and experimental animal models to substantiate the carcinogenicity of ethanol since this toxin is classified in group 1 [28]. Nonetheless, a very low frequency of neoplastic alterations is observed in long-term intervention in rats [178]. In mice, a statistical trend was observed in 2-year-long ethanol intervention in drinking water regarding the incidence of neoplastic lesions, mainly adenomas [179]. Alcohol is usually applied as a promoting or co-carcinogenic agent in chemically induced HCC models in drinking water or as a part of liquid diets (Table 4). Nevertheless, the experimental use of ethanol as a promoter of DEN-initiated models displays controversial results in rats. The cessation of long-term alcohol administration after DEN initiation seemed to enhance GST-P+ foci development. However, intermittent alcohol intake showed to decrease in the number of these preneoplastic lesions. As ethanol exerts suppressing effects on liver regeneration, the cessation of long-term alcohol insult may reactivate hepatocyte proliferation, thereby promoting preneoplastic liver development [180,181]. As these effects were not evaluated in neoplastic lesions and the exact mechanisms were not fully evaluated, a model-dependent effect should not be discarded. Conversely, using the same model, ethanol significantly increased the Ki-67 positivity in GST-P+ foci and incidence/multiplicity of HCC in Cx32 dominant-negative transgenic rats but not in wild-type counterparts. In addition, increased nuclear-phosphorylated Erk1/2 and reduced Erk1/2-inhibitor Dusp1 protein and mRNA were only observed in Cx32 transgenic rats, suggesting enhancing effects of ethanol on DEN-induced hepatocarcinogenesis via Cx32 dysfunction, which is commonly observed in human chronic liver disease [182].

Ethanol also elevates the abundance of preneoplastic and neoplastic lesions induced by an RH model in Sprague Dawley rats [184] and the 2-amino-3, 8-dimethylimidazo [4,5-f] quinoxaline (MeIQx) carcinogen, a relevant heterocyclic amine found in cooked meat [183,188] (Table 4). In the RH model, a 5% ethanol intervention for 15 weeks enhanced the size and area occupied by GST-P+ preneoplastic foci and the multiplicity of neoplastic lesions while not significantly altering their incidence (Table 4). Furthermore, ethanol increased the proportion and the multiplicity of preneoplastic foci with the double expression of GST-P and transforming-growth factor-alpha (TGF-α) markers, indicating that TGF-α may be a pathway for the promoting activity of ethanol towards hepatocarcinogenesis [184] (Figure 4B). In addition, high doses (10% and 20%) of ethanol dose-dependently increased the incidence and/or the multiplicity of hepatocellular adenoma/carcinoma induced by MeIQx in rats [183]. A low dose intervention increased the number of small MeIQx-induced GST-P+ foci by enhancing proliferating cell nuclear antigen (PCNA) immunohistochemical staining and the levels of 8-hydroxydeoxyguanosine, a marker of oxidative DNA damage [188]. Similar effects on the enhancement of GST-P+ foci emergence were also observed when ethanol was co-administered with MeIQx or N-nitrosomorpholine [189,190].

In mice, the promoting effects are similar to the rat bioassays. In general, alcohol is proposed to increase the multiplicity and/or size of both preneoplastic and neoplastic lesions in DEN-induced models while not having pronounced effects on the incidence of lesions compared to DEN (pair-fed or not)-only counterparts (Table 4). Brandon-Warner et al. [186] reported a slight but significant increase in the number and area occupied by preneoplastic foci mediated by a 10/20% 8-week-long ethanol intervention after multiple DEN administrations in juvenile B6C3F1 mice. Promoting effects on the size of macroscopically-identified neoplastic observed in a 5% ethanol 10-week-long intervention in adult C57BL/6 mice submitted to multiple DEN injections [191]. Likewise, Mercer et al. [185] described an increase in the mean number of lesions, and, specifically, in the number of eosinophilic cell foci and adenomas after a 5% ethanol 16-week-long intervention in a neonatal mouse model. In this case, a significant ethanol-induced enhancing effect on incidence was observed only concerning eosinophilic foci phenotype (Table 4). Mechanistically, the alcohol-promoting effects in DEN-initiated mouse models are related to immune system disturbances, oxidative stress, and sustained cell proliferation hallmarks (Table 4). Ethanol intervention promoted the mRNA expression of epithelial-mesenchymal transition (EMT) biomarkers, such as E-cadherin, Snail, MMP-9, and also favored M2 polarization of tumor-associated macrophages by upregulating IL-4, IL-10, CD206, CXCL2, and CCL22, and downregulating IL-12 mRNA (Figure 4B). Antitumoral CD8+ T cells were decreased in the liver of ethanol-fed mice as well [191]. Ethanol exacerbated DEN-induced oxidative stress in the liver by enhancing malondialdehyde while diminishing glutathione levels [186]. Of note, ethanol-mediated increase in hepatocyte proliferation was associated with a cytoplasmic β-catenin staining pattern in alcohol-associated HCC cases in humans [192], and the ethanol-mediated activation of the β-catenin axis was also implicated in the increase of stemness and metastasis of HCC cells [193]. Likewise, increased expression of Wnt7a, β-catenin, phosphorylated GSK3β, and several targets of Wnt/β-catenin pathway, such as glutamine synthetase, cyclin D1, Wnt1 inducible signaling pathways protein (WISP1), and matrix metalloproteinase-7, were detected in the liver of male Sprague-Dawley rats fed only alcohol for ~5 months via total enteral nutrition [185]. Although the described molecular events are directly correlated with liver tumorigenesis, most investigations did not perform molecular analysis in neoplastic lesions but only in whole liver samples. In a recent investigation that considered the epidemiological evidence of the synergistic effects of alcohol with other common HCC risk factors, Ma et al. [187] showed that the combination of HF and ethanol accelerates by 2-fold or 8-fold the DEN-induced HCC incidence in mice compared to HF-fed only counterparts (Table 4). Further experiments in IL-17 KO mice showed that the expression of many classical tumorigenic genes in HCC, as well as inflammatory, fibrogenic, and lipogenic genes in non-tumoral tissue, is IL-17-dependent. Ethanol-induced IL-17 signaling in steatotic hepatocytes, mediated by Th17 cells and macrophages, promoted (1) lipogenesis via activation of the caspase-2-SP1-SREBP1/2-DHCR7 pathway and (2) hepatocellular damage by preventing TNFR1 exocytosis [187] (Figure 4B).

### 2.4. Genetically Engineered Mouse Models

#### 2.4.1. Hepatitis Virus Transgenic Mice

First established in the 1990s, HBV and HCV transgenic models enabled a deeper insight into the pathogenetic mechanisms involved in viral-induced chronic liver injury and malignant transformation. Using transgenic technology, the transgenic phenotype is intentionally obtained by the insertion of exogenous viral DNA. To investigate the oncogenic mechanisms involved in HBV infection, transgenic mice expressing different components of the viral particle were developed (Table 5). Kim et al. [194] first introduced transgenic mice containing the entire of HBx protein-coding gene and its transcriptional enhancer via micro-injection into single-cell embryos of the CD1 mice strain. Further studies were also performed in C57BL/6xDBA and C57BL/6xCBA hybrid backgrounds [195,196]. The HBx protein acts as a transcriptional transactivator, promoting the expression of many oncogenes, including FOS, JUN, and MYC, also inducing HBV transcription and replication [197,198]. Most HBx-transgenic mice feature macro and micro fatty changes at 6–11 months of age. Although inflammation was not frequently observed from 6–15 months of age, most mice developed preneoplastic liver dysplasia and adenomas from 6–11 months [196]. Proteomic analysis of these preneoplastic stages revealed that 22 proteins were differentially expressed in dysplasia or adenomas, 5 upregulated and 17 downregulated. Deregulated proteins indicated that alterations in glycolysis and lipogenesis might be critical during the early (preneoplastic) stages of HBx-induced hepatocarcinogenesis, considering the upregulation of fatty acid-binding protein 2 (FABP2) and the downregulation of cytoplasmic malate dehydrogenase (MDH) and mitochondrial 3-ketoacyl-CoA thiolase (HADHA). These alterations were validated in human HCC samples. In addition to metabolism-related proteins, the authors found that raf kinase inhibitory protein (RKIP), a negative regulator of Raf-1, was downregulated at all hepatocarcinogenesis stages [196].

Another transgenic strain called Tg (Alb-1 HBV) Bri44 was designed to contain coding regions for HBx, HBsAg, and pre-S proteins (Table 5). These mice feature a stepwise liver disease with prolonged liver cell injury, death, pronounced inflammation, and elevated compensatory hepatocyte proliferation, which is triggered by the increased production and retention of the HBV large envelope polypeptide. The necro-inflammatory context resembling human disease leads to the progressive development of preneoplastic foci, adenomas, and carcinomas [199,200,210]. Barone et al. [211] revealed the early molecular events in 3 -week-old Tg (Alb-1 HBV) Bri44 mice by transcriptomic analysis. It was found that 25 genes are upregulated, including those involved in NF-κB signal transduction (*Vcam1* and *Cxcr4*), regulation of transcription (*Hmgb2*, *Nfatc1*, *Nupr1*, and *Atf3*), cell cycle and proliferation (*Cdkn2d* and *Slfn2*), and negative regulation of apoptosis (NuprI), and 20 downregulated, such as anti-proliferative (*Ghr* and *Erbb3*) and pro-apoptotic (*Bnip3*) genes. As a long latency time is required for Tg (Alb-1 HBV) Bri44 mice to develop neoplastic lesions (Table 5), protocols using DEN or AFB1 may synergistically act with the genetic modification to accelerate neoplastic lesion development, presenting an incidence of 75–90% within 15 months [212]. Lai et al. [201] developed a transgenic mouse model featuring a mutated HBV pre-S/S gene and its promoter in C57BL/6 mice, since rtA181T/sW172* mutation confers resistance to antiviral therapies, exerting an oncogenic potential [213]. Two transgenic strains were developed, both featuring an sW172* mutation but one expressing high and the other low intrahepatic levels of HBV surface antigen (HBsAg). Although the incidence of HCC was low in both strains after 18 months of age (Table 5), enhanced ER stress-related and proliferation-related proteins were increased in the non-neoplastic tissue. The investigation of miRNA-mRNA networks further revealed that transgenic mice also presented microRNA-873-mediated reduced expression of tumor suppressor CUB and Sushi multiple domains 3 (CSMD3) protein.

Concerning HCV, mice expressing the full core gene presented progressive morphological and molecular changes that ultimately resulted in the development of HCC. At 9 and 12 months of age, the mice only showed pronounced steatosis without inflammatory or neoplastic lesions. At 16 months, the major neoplastic changes were eosinophilic adenomas with fatty changes, while between 16 and 19 months of age, some nodules were adenomas or well-differentiated HCCs [202] (Table 5). Proteomic analysis stepwise of this model revealed that at 12 months, proteins related to respiration, electron-transfer system, apoptosis, fatty acid metabolism defense against oxidative stress were upregulated. At 16 months, most differentially expressed proteins were downregulated, including those involved in anti-oxidant defense, β-oxidation, and apoptosis [214]. In another model of mice expressing HCV core-E1-E2 proteins treated with DEN, the genetic modification increased the size of neoplastic alterations, increasing cell proliferation and decreasing apoptosis [215]. Although HBV and HCV transgenic models are usually time-consuming (long latency time for HCC emergence), their main advantages comprise the investigation of virus-related oncogenic mechanisms and the screening of therapeutic options.

#### 2.4.2. Other Gene Expression Systems

A myriad of genetic manipulations to assess the effects of the activation of oncogenes and/or disabling of tumor suppressor genes have been developed in mice in the past decades. As the stepwise hepatocarcinogenesis process involves the acquisition and accumulation of genomic alterations, these models enable the investigation of potential therapeutic targets in preclinical settings. The methodological approach consists of the use of an albumin promoter, the induction of specific genes with molecules, as well as the recently developed hydrodynamic tail vein injection (HTVI) using the Sleeping Beauty (SB) transposase system or CRISPR/Cas9 genome editing tool (Table 6). Developed by Sandgren et al. [216], transgenic mice overexpressing the oncogene MYC, directed by the albumin enhancer/promoter or alpha1-antitrypsin promoter technologies, displayed mild to severe hepatic dysplasia in young mice, while hepatic neoplasia emergence required a long latency time, up to 16 months (Table 5). MYC overexpression leading to tumor development is also achieved by the integration of the Woodchuck Hepatitis Virus (WHV) in the mouse genome [217]. In this model, the overexpression of MYC along with IGF-2 during the neonatal stage, driving a strong proliferative stimulus, is proposed to drive hepatocellular transformation [218]. Frequently found in the corresponding human disease, activating β-catenin gene mutation is featured in 50–55% of WHV or promoter-activated MYC -driven liver carcinogenesis [219].

Double transgenic mice bearing both albumin enhancer/promoter c-myc and metallothionein 1 promoter TGF-α were developed by Murakami et al. [230] and Santoni-Rugiu et al. [203]. The co-expression of both genes in the mouse liver promoted neoplastic lesion emergence compared to MYC overexpression alone (Table 5). Increased levels of TGF-α mRNA and protein during the early stages of liver carcinogenesis in MYC/TGF-α transgenic mice are proposed to have a key role in the clonal expansion and malignant conversion of the preneoplastic cell population [230]. HCC in c-myc/TGF-α transgenic mice had extensive genomic instability (loss heterozygosity) while displaying a low rate of β-catenin mutation and subsequent nuclear accumulation (12.5%) [205]. As MYC/TGF-α transgenic mice showed increased expression of oncogenic E2F1 and 2 and induction of their target genes [203], E2F-1 and double MYC/E2F-1 transgenic mice were developed [203,205]. Compared to MYC or E2F-1 transgenic mice, double transgenic animals also showed potential cooperation between MYC and E2F-1 oncogenes (Table 5), featuring a high frequency of β-catenin mutational activation and nuclear accumulation in both adenomas and carcinomas [205]. Based on comparative analysis of the transcriptome of these models with the corresponding human disease, Lee et al. [76] proposed that MYC, E2F-1, and double MYC/E2F-1 transgenic mice had similar global gene expression to a group of human HCCs with better survival, whereas MYC/TGF-α transgenic mice reflected a poorer survival HCC group. Regarding the frequently targeted Wnt/β-catenin pathway, a mutant mouse strain displaying adenomatous polyposis coli (*Apc*) deletion by the injection of adenovirus encoding Cre recombinase led to the development of HCC albeit presenting low incidence [207] (Table 5). On the other hand, simultaneous mutations in the *Catnb* (β-catenin) and *Hras* genes by using the same technology cooperatively accelerated HCC development (Table 5), as β-catenin may promote the clonal expansion of *Hras*-induced dysplastic (preneoplastic) cells [208]. More recently, using HT coupled with the SB transposon system, Chung et al. [209] developed a mouse strain transfected with transposons expressing MYC and a short hairpin RNA downregulating p53. Interestingly, tumor incidence and multiplicity were accelerated by the establishment of a CCl_4_-induced fibrosis context. Employing the same system, Tao et al. [227] modeled the concomitant HMET overexpression or activation and CTNNB1 mutations found in 9–12.5% of human HCCs by developing hMet/β-catenin point mutant mice. HT method to deliver a CRISPR plasmid DNA expressing Cas9 models targeting both tumor suppressor genes *Pten* and *Tp53* has also been recently employed [231,232], underscoring the potential of this tool for the development of novel hepatocarcinogenesis models.

### 2.5. Humanized Mouse Models

HCC is an inflammation-driven cancer, and recognition of the involvement of components of the innate and adaptive immune system in the development and progression of HCC has accelerated research into new therapies capable of targeting and/or modulating the immune system [233]. Promising results of combining current HCC treatment modalities, such as locoregional treatment and anti-angiogenic therapy combined with immunotherapy, have resulted in recently approved new treatment strategies for HCC [234,235]. Importantly, preclinical translational research with the ultimate goal of demonstrating therapeutic efficacy with an acceptable safety profile for human disease requires essential components that characterize human (patho)physiology. In HCC, this relates to neoplastic hepatocytes surrounded by a tumor micro-environment (TME) composed of (suppressive) immune cells. Currently used HCC models in immunocompetent mice do not accurately represent the human immune system since significant immunobiological differences exist between mice and humans, including dissimilarities in T cell signaling, immune cell receptor expression, and antigen presentation [236,237]. Xenograft models require immunocompromised mice to avoid rejection of an implanted human cell line or patient-derived material and thus do not provide a solution for immune-oncology studies [16]. More recently, mice harboring a humanized immune system (HIS) have been developed and now opened the field of research towards the use of preclinical models based on patient-derived HCC tissue in the context of an effective human immune system, essential in the evaluation of immune-oncology drug efficacy and safety [16,238]. In addition to the humanization of immunocompetent mice via transgenesis, immunocompromised mice can acquire a humanized immune system through engraftment with human peripheral blood mononuclear cells (PBMCs) or CD34+ hematopoietic stem cells (HPSC) [239].

In the PBMC-humanized mouse model, PBMCs from healthy donors are intravenously, intraperitoneally, or intrasplenically injected within several days after implantation of a human cell line or patient-derived xenograft, CDX and PDX, respectively, in an immunodeficient mouse [240,241]. Despite comprising several types of immune cells of both the lymphoid and myeloid lineage at transplantation, PBMC engraftments give rise to an almost exclusively T cell-oriented humanized immune system, since appropriate signals for the survival and expansion of B, NK, and myeloid cells are lacking [240,242]. The engrafted lymphocytes are functionally mature, and HIS mice can be utilized almost immediately after PBMC injection for therapy evaluation. However, in addition to its restriction to the evaluation of T cell-based immunity, the use of this model is limited to short-term experiments because injection of PBMCs elicits xenogeneic graft-versus-host disease (GvHD) a few weeks after engraftment [239]. Delay of GvHD development and improvement of the overall immune functionality can be obtained through genetic enhancements of the recipient mice, including replacement of murine major histocompatibility complex (MHC) by human leukocyte antigen (HLA) expression [242]. A new mouse strain lacking murine MHC molecules (NSG-(KbDb)null(IA)null) has been created in which human PBMCs can be engrafted without the development of acute xenogeneic GvHD [243]. The model has effectively been used to evaluate immune checkpoint inhibitors in human cancer xenograft models and is ideally suited to evaluate anti-cancer (immuno)therapy [244]. Despite being relatively straightforward, fast, and cost-effective compared to other HIS models, the PBMC-humanized mouse model has thus far only been applied as the HCC CDX model [245,246]. As an alternative to PBMCs engraftments, CD34+ HSCs derived from multiple potential sources, including granulocyte colony-stimulating factor (G-CSF)-mobilized peripheral blood, adult bone marrow, fetal liver, and umbilical cord blood can be engrafted in immunocompromised mice [239,241].

The CD34+-humanized mouse model has the advantage of displaying a more complete representation of the human immune system. However, due to the lack of human cytokines and growth factors, the developed T, B, NK, and myeloid cells all exhibit functional impairments [247]. Transgenic immunocompromised mouse strains expressing human cytokines and growth factors could further enhance the engraftment of CD34+ HPSC and support the development of functional human immune cells. In this respect, transgenic expression of human IL-3 and granulocyte-macrophage colony-stimulating factor (GM-CSF) in NOD/Shi-scid IL2rγnull (NOG) mice is beneficial for the development of myeloid cells [240]. Importantly, since human T cells derived from engrafted HSCs undergo a positive and negative selection on murine MHC molecules during development in the thymus, tolerance towards the murine host is established [248]. However, as murine thymic epithelial cells do not express HLA, the resultant T cells are not able to recognize antigens in an HLA-restricted manner. Consequently, these HIS mice elicit an inappropriate T cell response against human HCC xenografts [249]. To overcome this issue, transgenic mice expressing HLA molecules matched to the PDX donor should be used [250]. To this end, Serra-Hassoun et al. [251] created a new lymphoid mouse strain which, in addition to the replacement of murine MHC by HLA, expresses human signal regulatory protein alpha (hSIRPα) on murine phagocytes to enable human HSC engraftment [251]. In contrast to the PBMC-humanized mouse model, which can be utilized almost immediately after PBMC engraftment, it takes up to 10 to 12 weeks to develop a robust humanized immune system following HPSC engraftment. Moreover, recipient mice need to be irradiated to ensure engraftment of human HPSC [239].

The success of HPSC engraftment is dependent on the age, sex, and strain of the mice, the route of engraftment, and the source of the CD34+ HPSC [239,247]. Despite having the advantage of enabling stable and long-term humanization and representing a substantial portion of the human immune system, notable limitations, such as incomplete immune cell development and the time required to establish the humanized immune system, have limited the utilization of this HIS model concerning HCC [242]. Hitherto, only Zhao et al. [19] effectively developed and used a PDX humanized HCC model for investigation of the human-specific TME and immunotherapeutic treatment strategies. In this model, 1 to 3 days old NOD-Prkdc-scid IL2rgnull (NSG) pups were irradiated and intrahepatically injected with human fetal liver-derived CD45+ HSCs. The created HIS mice were subcutaneously transplanted with an HLA-matched PDX 8 to 10 weeks after HPSC engraftment. Effective human immune responses and the therapeutic efficacy of immune checkpoint inhibitors were demonstrated [19]. Since the HCC tumor interacts with both infiltrating immune cells and liver-resident cells, orthotopic implantation of a PDX in the hepatic micro-environment of CD34+-humanized mice would represent an even more attractive platform for preclinical evaluation of immunotherapeutic and other HCC treatment strategies but remains to be developed.

A final HIS model that has not yet found its way to the field of HCC research is the bone marrow/liver/thymus (BLT) model. In this model, fetal liver and/or bone marrow-derived CD34+ HSC engraftment is preceded by transplantation of a small fragment of human fetal thymic and liver tissue under the kidney capsule of immunocompromised mice [242]. As it provides human thymic tissue, the BLT model enables improved human T cell development. However, since the positive selection of T cells occurs solely on human MHC molecules and T cells with an affinity for murine MHC are not eliminated, the incidence of xenogeneic GvHD is higher in this model compared to the CD34+- model [247]. This problem could be overcome by using a transgenic MHC-deficient mouse strain. The technical difficulty of creating the mice and the necessity of human fetal tissue are 2 major factors that currently impede the utilization of the BLT model over the PBMC or CD34+-humanized mouse model [242].

## 3. In Vitro Models of HCC

### 3.1. Primary Hepatocytes

Because of their high resemblance to the in vivo phenotype, primary human hepatocytes (PHH) (Table 7) are considered the gold standard for in vitro studies on biotransformation, toxicity and drug-induced liver injury [252,253,254,255]. The liver is a major target for chemical-induced injury caused by carcinogens [256]. The application potential of primary hepatocytes from rodent or human origin in HCC studies focuses on the initiation and promotion stages of cancer [254,256,257]. In this respect, PHH have been used for genotoxicity assays, albeit to a lesser extent compared to their rodent counterparts [254,256,257]. More recently, PHH have been used to elucidate the molecular pathways or genomic effects in response to carcinogens [246,258,259,260,261,262]. In addition, PHH have been addressed to evaluate the molecular and cellular events of HCC initiation through in vitro transformation based on lentiviral-transduction of oncogenic Harvey-RAS, simian virus 40 (SV40) small T antigen, and SV40 large T antigen [263]. The major drawbacks of PHH are the high donor-to-donor variability [253] and their progressive dedifferentiation, which makes them less suitable for long-term culture [264]. This dedifferentiation process is already initiated during the isolation process [265]. To overcome dedifferentiation, sandwich cultures or spheroid models of PHH can be used [261,266,267,268,269]. Although spheroid models of PHH have not been used in genotoxicity studies, sandwich cultures of PHH have been applied to elucidate the epigenetic effects of AFB1 in HCC initiation [262] and to assess the modulation of AFB1-mediated genotoxicity by chemopreventive chemicals [270].

### 3.2. Hepatic Cell Lines

Cell lines (Table 8 and Table 9) are popular models for drug screening studies [256], drug safety testing [176], and studies related to liver disease [253] due to their ease of use, phenotypic stability and reproducibility. Human liver cell lines are typically immortalized by genetic engineering or selected based on their tumorigenic phenotype, implying they are derived from human tumors [253,274]. Upon genetic engineering, PHH are most commonly transfected or transduced to overexpress human TERT or viral oncogenes, such as SV40 large T antigen or human papillomavirus16 E6/E7 genes [317]. Immortalized cell lines are often used in parallel with tumor-derived cell lines to assess side effects and therapeutic effects of various compounds [275,318,319,320] or in studies pinpointing molecular, genetic and epigenetic alterations in HCC [74,276,277,321]. In addition, immortalized cell lines are used to study the influence of tumor micro-environments and overexpression of genes leading to cancer initiation [322,323]. Tumor-derived cell lines are the most widely used cell lines in HCC research [256,324]. Indeed, they are harnessed to assess cancer characteristics, such as cell proliferation, migration, metastasis, evasion of cell death and invasion [278,281,325,326,327,328,329]. Moreover, liver cancer cell lines are frequently used for elucidating molecular mechanisms of HCC during overexpression or silencing gene studies [282,326,327,329]. Liver cancer cell lines can be equally used to provide insight into genetic changes of HCC [277], for the identification of new drug targets [326], and for testing anti-cancer therapies [325,326,330,331]. However, their clinical relevance for the latter application may be questioned [279]. Since each cell line is derived from a single donor, they fail to represent the well-known intertumor and intratumor diversity that hampers the development of HCC therapies [256,279]. This flaw can be circumvented by using several cell lines in parallel [274,324]. Based on their genetic characteristics, a panel of cell lines could be selected to represent different HCC subclasses [274,277,324]. Although more frequently used to elucidate HCC biology and progression, liver cancer cell lines are also employed in genotoxicity testing [254,256,277]. The majority of genotoxicity studies are performed in mouse lymphoma cells, human lymphoblast cells, or Chinese hamster lung cells, which are metabolically incompetent, making their human relevance questionable [254]. In fact, p53-deficient rodent cell lines give rise to more false positives compared to human cell models [332]. Therefore, the use of human liver cell lines in genotoxicity/mutagenicity assays is gaining increasing attention [254]. An emerging tool includes the human hepatoma HepaRG cell line [333]. In contrast, compared to HepG2 cells, which are by far the most widely used cells in liver studies, HepaRG cells functionally express biotransformation enzymes at a level that is comparable to PHH and are therefore a more sensitive cellular system for (geno)toxicity testing [256,333,334,335]. In addition, HepaRG cells have been shown valuable for understanding the mechanism of action of mutagenic/carcinogenic compounds [336].

### 3.3. Co-Cultures

As much as 70–80% of the human liver consists of hepatocytes, while 5–6% comprises non-parenchymal cells such as Kupffer cells, hepatic stellate cells (HSC), and sinusoidal endothelial cells [345,346]. To mimic this in vivo cellular heterogeneity, co-cultures can be set up in which PHH [347,348] or liver cell lines are seeded together with non-parenchymal liver cells, such as HSC [282], endothelial cells [281,349], stem cells [292], or immune cells (Table 7) [283]. In such co-culture settings, transwell chambers can be used, allowing communication between both cell populations [350]. Co-cultures of hepatic cell lines with tumor-specific cells, like cancer-associated fibroblasts or tumor-specific neutrophils [285], provide in vivo-like tumor-specific microenvironments [284,285]. This leads to a more relevant in vitro model that is capable of manifesting the changing cancer characteristics of HCC cell lines under the influence of other cell types [351]. Moreover, co-culture systems provide a valuable model in immune cell therapy studies and research concerning crosstalk between HCC cells and the immune system. In this respect, co-cultures of HepG2 cells with natural killer cells display anti-proliferative effects, and the addition of M1 macrophages reduces HCC viability, invasion, therapy resistance, and migration, whereas M2 macrophages promote tumor invasiveness in HCC co-cultures [283,352,353]. Co-culturing HepG2 cells with endothelial cells yield an in vitro system appropriate for studying angiogenesis [281].

### 3.4. Stem Cell-Derived Models

Several studies have indicated that HCC develops from cancer stem cells (CSC). CSC are self-renewing and give rise to the different cell lineages in HCCs. Since CSC possess the capacity to form tumors, they are the major drivers of chemotherapeutic resistance, metastasis, and post-treatment tumor recurrence [354]. In this respect, understanding the malignant reprogramming that occurs in these cell types is crucial for the development of effective HCC therapies [355,356]. Induced pluripotent stem cell technology is an ideal tool to model the reprogramming that leads to tumorigenesis and cancer progression (Table 7). Induced pluripotent stem cells are even regarded as a potential therapy for HCC treatment by targeting CSC-related genes. Induced pluripotent CSC can be generated from liver cancer cells to specifically model liver CSC [286]. As such, various liver cancer cell lines have been reprogrammed via retroviral particles, introducing 4 stem cell transcription factor genes, namely KLF4, Sox2, Myc, and Oct4, to better understand the reprogramming process [356]. Together with NANOG and LIN28, these transcription factors have been detected in HCC and are associated with negative clinical outcomes. Besides induced pluripotent CSC, cells with stemness properties are also extracted from primary tumor material or cell lines to be used in studies that evaluate the molecular and epigenetic mechanisms of HCC [357]. Furthermore, stem cells are not only used to model tumorigenesis but also to test patient-specific direct reprogramming therapies and to identify molecular targets for blocking tumor initiation [358,359,360].

### 3.5. Spheroid and Organoid Models

While 2D models are valuable tools in liver studies, they lack in vivo-like cell density and a complex microenvironment [253]. 3D models (Table 7), such as spheroid cultures and organoid cultures, have been developed to overcome these flaws. Spheroids can be derived from PHH and hepatic cell lines [361]. Most liver cell lines are less differentiated compared to PHH, which is mainly reflected by a lack of biotransformation capacity [176,253,256]. Throughout the years, 3D spheroid models have been introduced to tackle this shortcoming [291,293]. As such, 3D spheroid cultures of human hepatoma HepG2 cells express more albumin and phase I and II biotransformation enzymes compared to 2D counterparts, making them more applicable for genotoxicity studies [290]. Besides genotoxicity assays, 3D spheroid models of liver cancer cell lines have been used for evaluating anti-cancer agents and drug sensitivity [294,362,363,364,365]. In addition, various studies have been set up in which 3D spheroid culture techniques are combined with co-culture approaches [292,295,296,348,365]. In this respect, a combination of primary HCC cells with extracellular matrix (ECM), endothelial cells, and fibroblasts in spheroid cultures creates a model with enhanced tumor-related and neo-angiogenesis markers to study potential HCC therapies [289]. Compared to unicellular 3D spheroid cultures, co-culture spheroid models display a more in vivo-like microenvironment, thereby creating a more pathologically relevant HCC model for drug-screening studies [365,366].

Organoids are self-renewing and self-organizing 3D tissues derived from various stem cell types, such as embryonic stem cells, induced pluripotent stem cells, organ-restricted adult stem cells, and primary tissue (Table 7) [297,298,299]. The difference with 3D spheroid models is that organoids contain various tissue-specific cells, which are all developed from the stem cell starting material through in vivo-like processes mediated by the provided ECM, namely Matrigel^®^ [367,368]. The latter is an extract of an Engelbreth-Holm-Swarm mouse sarcoma that contains many ECM proteins and some less defined biochemical signaling molecules, including growth factors, necessary for the development of organoids [367,368,369,370]. This model can mimic the functionality and architecture of native liver tissue [298]. Liver organoids can be vascularized when hepatic endoderm cells directly differentiated from induced pluripotent stem cells are co-cultured with endothelial and mesenchymal cells [371]. Since the model is based on primary healthy or tumorigenic material, organoids create new possibilities for regenerative medicine, personalized drug discovery, toxicity studies, and gene therapy [297,300]. Liver organoids have been used to model liver cancer initiation [299,372], to study HBV-related hepatocarcinogenesis [301], to investigate drug sensitivity on patient-derived models [302] and to perform molecular and cellular characterization of HCC [299]. Patient-derived liver organoid models permit the generation of tumor biobanks that enable the profiling of genomic diversity as well as drug sensitivity studies [367]. Furthermore, they can not only assist in the assessment of genomic intertumor diversity but equally of intratumor diversity, allowing the study of variations in drug responses within one single tumor [303].

### 3.6. Precision-Cut Liver Slices

Precision-cut liver slices from rat, mouse, or human origin are ex vivo tools that retain the complex native liver environment containing interactions between all liver cell types and the ECM (Table 7) [309,310,311]. Liver slices are usually 100–250 µm thick and have a diameter around 5 mm allowing nutrients and oxygen to easily be diffused across all cell layers [312]. Although precision-cut liver slices have been used for various purposes, their use in HCC studies is rather limited. Precision-cut liver slices prepared from primary tumor material have been used as a model to study anti-cancer drugs and oncolytic virotherapy [313,314,373]. It has been suggested that precision-cut liver slices could represent a valuable model to evaluate patient-specific drug responses and therapy resistance in addition to predicting side-effects on adjacent healthy tissue [314,374].

## 4. Therapeutic Relevance of the HCC Models

The treatments conventionally used in HCC patients, such as tumor resection, chemotherapy, radioembolization, and liver transplantation, are highly dependent on the cancer stage. In advanced-stage HCC, these procedures become unfeasible, requiring the use of systemic therapies [375,376]. However, HCC cells show high resistance to conventional chemotherapy. Sorafenib, a multi-tyrosine kinase inhibitor that reduces tumor growth and angiogenesis, was developed in 2008. Despite this drug having prolonged patient survival in a few months [377], its clinical use has been limited to adverse side effects and refractory drug response, mainly associated with genetic heterogeneity of HCC [378,379,380]. Recently, novel classes of multi-tyrosine kinase inhibitors have been developed, such as lenvatinib, regorafenib and cabozantinib; however, the prognosis of HCC patients is still poor [381,382,383]. This scenario urgently drives the search for new therapeutic targets, drugs and therapies, such as immuno and gene therapies [384]. From sorafenib to immunotherapy, in vivo and in vitro models have shown fundamental importance in the pre-clinical phase for anti-HCC therapies.

The use of syngeneic and xenographic models has been widely used in studies of the combination of drugs used in HCC therapy, such as sorafenib, and drugs that enhance its effects by reducing tumor resistance to treatment [272,385,386,387], in immunotherapeutic studies [273,388,389,390] and new drug trials [391,392]. In the context of the combination of drugs with known potentialities, chemical induction of HCC by DEN in Fisher and Wistar rats (50 mg/kg, once a week, for 12–14 weeks) was used respectively to evaluate the effects of treatment with sorafenib + ARQ (AKT inhibitor) and sorafenib + fluvastatin (cholesterol-lowering), showing that the combination of drugs increased apoptosis, reducing cell proliferation, angiogenesis and activation of HSC [393,394]. The DEN-induced models have also been applied in mice to test new drugs, such as Romidepsin, a histone deacetylase inhibitor, cell cycle inhibitor and apoptosis inducer [395]. The ability to induce HCC as a late event of CCl_4_ induction was used in mice (16% in corn oil, 3x/week/8 weeks) to evaluate the treatment of Sorafenib+ MAPK/ERK pathway inhibitors [396]. On the other hand, studies also associate the carcinogenic action of DEN with the fibro-cirrhotic capacity of CCl_4_ in the chemo [397] and immunotherapeutic tests [398]. TAA has also been used in preclinical tests in mice (200 mg/Kg ip or 200 mg/L in drinking water) associated or not with syngeneic and xenographic models, enabling the evaluation of drugs with known or promising potential, chemo or immunotherapy [386,388,399].

Diet is one of the factors influencing the development of HCC, and models that combine STZ and high-calorie diet to mimic HCC resulting from the late stages of NAFLD/NASH are commonly crucial tools in the prevention and treatment of this process. The preclinical use of this model in mice in the evaluation of Liroglutide, a Glucagon-like peptide-1 (GLP-1) receptor used to control glycemia, observed improvement in NASH and suppression of hepatocarcinogenicity [400]. Furthermore, Berberine, known for the treatment of gastroenteritis and for its promising anticancer potential, in this model, reduced tumorigenesis, angiogenesis and inflammation [401]. Genetic engineering enabled the creation of transgenic animals with structural and metabolic alterations totally directed to the specificity of the questions formulated for the object of study. In the preclinical researches of HCC, these animals can be inserted in different models, directing the results to a specific signaling pathway both in the testing of new drugs [386,388,396,397] and in new therapies [388,389,390,398].

Humanized models allow, for example, artificially created human antibodies to prove their efficiency in humanized mice, increasing the reliability of the clinical trial results. Bi and collaborators constructed a bispecific antibody for GPC3/CD3 and tested the antitumor activity in several cell lines Huh-7, HepG2, Hep3B, SK-Hep-1, and SK-Hep-1-GPC3 and in a xenographic model of subcutaneous inoculation of Huh7 cell line in NOD-SCD mouse. The results not only proved the efficient destruction of CPC3 positive cells but also proved that CPC3 is not present in normal cells and can be an HCC-specific antigen and, therefore, an excellent therapeutic target [390]. Currently, preclinical studies combine numerous tools such as liver cell lines, syngeneic and xenographic models, chemical induction and transgenic animals [386,388,393,396,397,399] in search of treatment alternatives for HCC.

## 5. Conclusions and Perspectives

Despite recent advances in HCC treatment, only 18% of patients survive more than 5 years after initial diagnosis, a percentage significantly lower compared to other cancer types [402]. The poor prognosis is usually attributed to late diagnosis and lack of response to adjuvant therapies [403]. The inefficiency of anti-neoplastic drugs can be attributed to the high molecular heterogeneity of HCC [404], which increases the need to identify new molecular targets based on signaling pathways activated in hepatocarcinogenesis according to etiologies. In addition, the low translational value of preclinical models could be directly associated with high rates of drug failure in human clinical trials [405]. Besides recapitulating the pathophysiology of liver cancer, the ideal model should be reliable, highly reproducible, technically simple, and at a low cost. The experimental modeling of HCC is particularly challenging due to the molecular heterogeneity and tumor microenvironment with a fibrotic and chronic inflammation background.

Next-generation sequencing has shown a high diversity of genetic and epigenetic alterations in HCCs, allowing the classification in subclasses according to their molecular signatures [13]. Several chemical-induced and/or diet-induced HCC models have been developed to induce all stages of hepatocarcinogenesis in rodents, which usually do not reproduce all molecular alterations observed in human HCC. The use of hybrid models combining classical HCC models and genetically engineered animals has been developed to overcome this critical barrier. More recently, the HTVI methodology has been applied to delivery transposon-based or CRISPR-Cas9 vectors to overexpress or delete/mutate tumor suppressor genes, respectively. This represents an innovative genetic manipulation method to unravel the role of cancer driver genes, specifically in hepatocytes. Furthermore, humanized PDX mice models have been proposed as a promising tool to study the immunological response in human HCC. This model opens new avenues to test novel immunotherapeutic targets and identify mechanisms of immune escape and resistance to immunotherapies. However, several technical limitations still need to be overcome. In vitro liver models have been extensively applied for toxicity studies and drug screening due to their relatively low cost and easy-to-do performance. In the last years, these models have progressively evolved from monolayer monocultures to highly complex 3D co-cultures to recapitulate the tumor micro-environment. In addition, recent advances in genetic manipulation can be easily applied to delete or overexpress target genes, develop new HCC cell lines, and reproduce the molecular heterogeneity of liver cancer in vitro.

**Figure 5:**
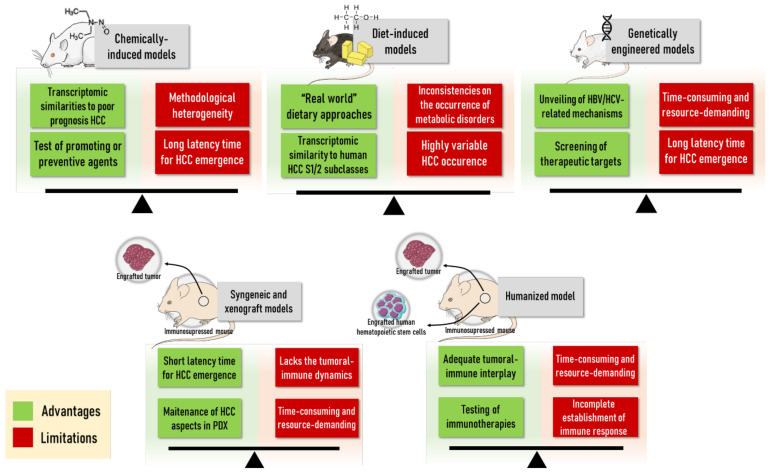
Main advantages and limitations of the main in vivo HCC mouse models available in the literature. The choice of the animal model must weight all the summarized aspects to provide relevant, translatable scientific data towards the understanding of hepatocarcinogenesis. The figure was composed with the aid of illustrations from the SMART-servier Medical Art available at https://smart.servier.com/ (accessed on 15 January 2021).

In the light of the spectra of in vivo and in vitro models available, and in order to provide a clearer understanding, their main advantages and limitations are summarized in Figure 5 and Table 7, respectively. The choice of a preclinical model must be a thoughtful and clearly defined process, weighting all the summarized aspects to provide relevant, translatable scientific data towards the understanding of hepatocarcinogenesis. In conclusion, the current in vitro-based and in vivo-based HCC models have shown several advantages and disadvantages according to the main application. Despite considerable advances in the HCC modeling, the lack of effective anti-neoplastic therapies urgently needs the establishment of more reliable, translational, and fast-induced HCC preclinical models.

## Figures and Tables

**Table 1:** Summary of some of the patient-derived xenograft HCC protocols in mice.

Model	Procedure	Animal (Species, Strain, Age)	Timepoints and Incidence of Lesions	References
Ectopic implantation of human HCC in mouse;	- Samples of human HCC were sectioned and inoculated on the dorsum of the mice; a region pretreated with anti-asialo GM1;	Female Balb/c athymic nude mice at 4-week-old;	- 1st and 2nd generation: tumor growth at the implantation site with 100% (6/6) of transplantability and size averaged 1.0 cm in diameter at 4th week; - 3rd generation: spontaneous liver metastasis at 12th week; - 4th generation: 100% (5/5) of significant liver metastases in the 4th week; - 8th week: all animals with transplanted tumors died with important metastases	[26]
LCI-D20: orthotopic Implantation of human HCC	- Tissue fragments measuring 2 mm^3^ of HCC from 30 human patients were implanted in the left hepatic lobe of the mice for 6 to 24 weeks;	Male BALB/cA nude mice at 4 to 6-week-old	- At 2nd week: initial liver metastasis with tumor colonies around the site of origin; - At 3rd: metastasis in mesenteric and iliac lymph nodes, hepatic hilum, mesentery and diaphragm; micrometastasis in pulmonary vessels; - At 6th week: generalized liver metastasis with vascular micrometastasis; micrometastasis in the lung parenchyma;	[22]
HCC cells	- Different human HCC cells were cultured and suspended in saline solution at 1.108 cells/mL; afterward, 20 µL of cell suspension was injected into the mice’s liver subserosa.	Male SCID at 6-week-old;	- At 6–7th week: the necropsy of the implanted animals was performed or before that period, if they showed signs of stress; - At 7th week: 2 of 12 cell lines had formed tumors only in the muscle; 5 of 12 cell lines had formed tumors in both muscles and lives; other 5 cells line did not form a tumor; - Intrahepatic metastases observed in 2 of 12 explored cells line (Li7; KYN-2), with the presence of neoplastic thrombi and new neoplastic sites distinct from the original or in the lobe that did not undergo implantation.	[23]
HCC-LY5 and HCC-LY10: Ectopic and orthotopic Implantation of human HCC in mice;	- Ectopic procedure: tissue fragments measuring 2 mm^3^ of HCC from human patients were transplanted in the subcutaneous tissue of the right flank of the mice. Tumor growth was measured once a week by palpation; when the tumor mass reached 10–15 mm, the tumor was removed, reimplanted in other mice, three more times; - Orthotopic procedure: fragments of ectopic models were implanted in the left lobe of nude mice;	NOD/SCID male and female mice and T cell-immunodeficient BALB/c-nu/nu mice at 6 to 8-week-old	- Ectopic implantation: - At 4th week: the necropsy of the implanted animals was performed. - 5 of 24 human HCC samples were transplantable (20.83%); - The growth rate of the tumor and the growth time increased according to the advance of the passages. Variable growth over 7–6 weeks (tumors with 2.7 mm^3^ to 7.2 mm^3^); - Orthotopic implantation: - At 6th week: the necropsy of the implanted animals was performed. - The rate of tumor formation was 100% (8/8).	[27]
Ectopic implantation from human HCC needle biopsies in mice	- 10 human HCC needle biopsies from patients were transplanted into the subcutaneous tissue of the mice	Nonobese, diabetic/severe combined immunodeficiency gamma-c mice at 10-week-old;	- 11 PDX models were established with 10 human HCC needle biopsies. - 4th to 28th week after implantation, it was the time necessary to observe tumor growth; - PDX subcutaneous injection of a biopsy cell suspension has a slow growth compared to intact tumor architecture; - Retransplanted tumors showed a shortened lag phase until the onset of tumor growth compared to the xenograft tumor derived from the biopsy tissue;	[24]

**Table 2:** Summary of some of the chemically induced hepatocarcinogenesis protocols in rats and mice strains.

Model	Procedure	Animal (Species, Strain, Age)	Timepoints and Incidence of Lesions	References
DEN	Single i.p., 90 mg/kg b.w.	Juvenile (5 weeks) C3H/He, DBA/2 and C57BL/6 mice (male)	- Adenomas: 0–20% at week 24, 10–50% at 36, and 20–50% at weeks 36–52 (strain-dependent) - Carcinomas: 0% at week 24, 0% at 36, and 0–40% at weeks 36–52 (strain-dependent)	[41]
	Multiple i.p. 1.5 or 3 mg/kg b.w. for 1 week (4×/week)	Juvenile (6 weeks) B6C3F1 and C3AF1 mice (male)	- Adenomas: 3–8% at weeks 100–120 (strain- and dose-dependent) - Carcinomas: 16–22% at weeks 100–120 (strain- and dose-dependent)	[42]
	Drinking water 15 mg/L for 3 weeks	Juvenile (4 weeks) B6C3F1 mice (male)	- Adenomas: 100% at week 24	[43]
	Multiple i.p. 25, 50, or 75 mg/kg b.w., for 4 or 8 weeks (1×/week)	Juvenile (4 weeks) C57BL/6 mice (male)	- Preneoplastic foci: 44–100% at week 33 (dose-dependent) - Adenomas: 0–33% at week 33 (dose-dependent) - Carcinomas: 0% at week 33	[44]
	Single i.p. 2.5, 10, 25 or 50 mg/kg b.w.	Infant (2 weeks) BALB/c mice (male)	- Adenomas: 7–87% at week 24 and 10–100% at week 40 (dose-dependent) - Carcinomas: 5–10% at both weeks at week 40 (dose-dependent)	- [45]
	Single i.p. 5 mg/kg b.w.	Infant (2 weeks) C3H/HeJ, B6C3F1 and C57BL mice (male)	- Adenomas: 90–100% at week 28 (strain-dependent)	[46]
	Single i.p. 1 mg/kg b.w.	Infant (2 weeks) C3H/HeJ, B6C3F1 and C57BL mice (male)	- Preneoplastic foci: 25–56% at week 22 and 46–100% at week 48 (strain-dependent) - Adenomas + Carcinomas: 25–67% at week 22 and 77–100% at week 48 (strain-dependent)	[47]
	Single i.p. 1 mg/kg b.w.	Infant (2 weeks) B6C3F1 mice (male)	- Preneoplastic foci: 75% at week 17 and 100% at week 22 - Adenomas: 0% at week 17 and 12.5% at week 22 - Carcinomas: 0% at both weeks 17 and 22	[48]
	Multiple i.p. 20 (1 dose), 30 (1 dose) and 50 mg/kg (6 doses) b.w., for 8 weeks (1×/week)	Infant (2 weeks) C57BL/6 mice (male and female)	- Adenomas + Carcinomas: 100% at week 24 - Obs.: Liver Fibrosis	[49]
	Gavage 80 mg/kg b.w. (weeks ~6–7)	Adult (~6–7 weeks) Sprague-Dawley rats (male)	- Carcinomas: ~5–20% at week 48 and 70	[50]
	Single i.p., 200 mg/kg b.w. (week 6)	Juvenile (4 weeks) F344 rats (male)	- Preneoplastic foci: 100% at week 42 - Adenomas: 7% at week 42 - Carcinomas: 0% at week 42	[51]
	Multiple i.p. 70 mg/kg b.w., for 10 weeks (1×/week)	Adult (6 weeks) Sprague-Dawley rats (male)	- Carcinomas: 100% at week 20 - Obs.: Liver Cirrhosis	[52]
	Multiple i.p. 30 mg/kg b.w. for 11 weeks (2×/week)	Juvenile (4–5 weeks) Sprague-Dawley rats (male)		[53]
	Multiple gavage 70 mg/kg b.w. for 14 weeks (1×/week)	Juvenile (4–5 weeks) Wistar rats (male)	- Carcinomas: 80% at week 30 and 100% at week 34 - Lung metastasis: 20% at week 34 - Obs.: Liver Cirrhosis	[54]
DEN and CCl_4_	- DEN: Single i.p. 10 mg/kg b.w. (week 2) - CCl_4_: Multiple i.p. 0.25 to 1.50 mg/kg b.w. for 8 weeks (3×/week)	Infant (2 weeks) C3H/HeJ mice (male)	- Preneoplastic foci: 100% at week 17 - Adenomas: 100% at week 17 - Carcinomas: 12.5% at weeks 17	[55]
	- DEN: Single i.p. 10 mg/kg b.w. (week 2) - CCl_4_: Multiple i.p. 0.2 mL/kg b.w. for 9 or 14 weeks (2×/week)	Infant (2 weeks) B6C3F1 mice (male)	- Preneoplastic foci: 100% at week 17 and 25% at week 22 - Adenomas: 37.5% at week 17 and 100% at week 22 - Carcinomas: 20% at week 17 and 50% at week 22	[48]
	- DEN: Single i.p. 200 mg/kg b.w. (week 4–5) - CCl_4_: Multiple gavage 0.5 mL/kg b.w. for 6 weeks (3×/week)	Juvenile (4–5 weeks) F344 rats (male)	- Preneoplastic foci: ~81% at week 16 - Adenomas: 100% at week 16 - Carcinomas: 73% at week 16	[56]
CCl_4_	- CCl_4_: Multiple i.p. 0.2 mL/kg b.w. for 9 or 14 weeks (2×/week)	Infant (2 weeks) B6C3F1 mice (male)	- Preneoplastic foci: 0% at week 17 and 12.5% at week 22 - Adenomas: 0% at week 17 and 12.5% at week 22 - Carcinomas: 0% at week 17 and 25% at week 22	[48]
DEN and TAA	- DEN: Multiple i.p. 20 (1 dose), 30 (1 dose) and 50 mg/kg (6 doses) b.w., for 8 weeks (1×/week) - TAA: Multiple i.p. 300 mg/kg b.w., for 4 or 8 weeks (2×/week)	Infant (2 weeks) C57BL/6 mice (male and female)	- Adenomas + Carcinomas: 100% at week 24 (for both doses)	[49]
	- DEN: Single i.p. 200 mg/kg b.w. (week 6) - TAA: Multiple i.p. 200 mg/kg b.w. for 24 weeks (2×/week) (cycles of 3 weeks of administration and 1 week of rest)	Adult (6 weeks) Wistar rats (male)	- Preneoplastic foci: 100% at week 26 - Adenomas: 30% at week 26 - Carcinomas: 10% at week 26	[57]
DEN, TAA and PB	- DEN: Single i.p. 200 mg/kg b.w. (week 6) - PB: 0.05% diet for 1 week - TAA: Drinking water 0.03% for 9, 10, 20 or 30 weeks	Adult (6 weeks) F344 rats (male)	- Adenomas: 0% at week 9, 16.7% at week 20, 100% at weeks 30 and 40 - Carcinomas: 0% at weeks 9 and 20, 25% at week 30, and 75% at week 40	[58]
DEN and PB	- DEN: Single i.p., 90 mg/kg b.w. (week 5) - PB: Drinking water 0.05% (week 7) for 17, 31 or 45 weeks	Juvenile (5 weeks) C3H/He, DBA/2 and C57BL/6 mice (male)	- Adenomas: 10–90% at week 24, 50–60% at 36, and 0–30% at weeks 36–52 (strain-dependent) - Carcinomas: 0% at week 24, 0–40% at 36, and 0–100% at weeks 36–52 (strain-dependent)	[41]
	- DEN: Gavage 80 mg/kg b.w. (weeks ~6–7) - PB: Drinking water 0.025, 0.05 or 0.1% (weeks ~7–8) for ~48 or ~70 weeks	Adult (~6–7 weeks) Sprague-Dawley rats (male)	- Carcinomas: ~5–20% at week 48 and 50–60% at week 70	[50]
	- DEN: Single i.p., 200 mg/kg b.w. (week 6) - PB: Drinking water 0.05% (week 7) for 36 weeks	Juvenile (4 weeks) F344 rats (male)	- Preneoplastic foci: 100% at week 42 - Adenomas: 64% at week 42 - Carcinomas: 50% at week 42	[51]
DEN, 2-AAF and PH	- DEN: Single i.p., 200 mg/kg b.w. (week 1) - 2-AAF: diet 0.02% for 2 weeks (weeks 3 and 4) - PH: 67% (week 3)	Adult Fischer 344 rats (male)	- Carcinomas: 68–71% at week 32, 75% at week 40 - Metastasis: ~3–4% at week 32, ~4% at week 40	[59]

i.p. = intraperitoneal; b.w. = body weight. 2-AAF: 2-acetylaminofluorene; CCl_4_: carbon tetrachloride; DEN: diethylnitrosamine; PB: phenobarbital; PH: partial hepatectomy; TAA: thioacetamide.

**Table 3:** Summary of some of the NASH-induced hepatocarcinogenesis protocols mice.

Model	Procedure	Animal (Species, Strain, Age)	Timepoints and Incidence of Lesions	References
HF/HS diet	High-fat and high sugar diet (~35% hydrogenated coconut oil and soybean oil, ~19% carbohydrate, *w/w*) for 12, 24 or 48 weeks	Juvenile (4 weeks) C57BL/6J mice (male)	Neoplastic lesions: 0% at weeks 12 and 24 and 20% at week 48	[165]
	High-fat diet (~21% fat and 0.1% cholesterol, *w/w*) and high sugar solution (23.1/18.9 g/L of fructose/glucose) for 56 weeks	Juvenile to adult (6–8 weeks) B6/129 mice (male)	Adenomas: 25% at week 56 Well-differentiated Carcinomas: 100% at week 56 Poorly-differentiated Carcinomas: 37.5% at week 56	[162]
	High-fat (23% *w/w*, of which 23% saturated, 34% trans, 31% monounsaturated (cis), 12% polyunsaturated) and high sugar solution (23.1/18.9 g/L of fructose/glucose) for 24 or 48 weeks	Juvenile to adult (6–8 weeks) B6/129 mice (male)	Neoplastic lesions: 0 at week 24 and 40% at week 48	[164]
	High-fat and high sugar diet (21.1% fat, 41% sucrose, and 1.25% cholesterol, *w/w*) and high sugar solution (23.1/18.9 g/L of fructose/glucose) for 12 or 24 weeks	Adult (9 weeks) C57BL/6J mice (male)	Neoplastic lesions: 0% at week 12 and 30% at week 24	[166]
HF/HS diet and CCl_4_	High-fat and high sugar diet (21.1% fat, 41% sucrose, and 1.25% cholesterol, *w/w*) and high sugar solution for 12 or 24 weeks CCl_4_: Multiple i.p. 0.2 mL/kg b.w. for 24 or 36 weeks (1×/week)		Neoplastic lesions: 0% at week 12 and 100% at week 24	
HF diet	High-fat (~24% fat, lard and soybean oil, *w/w*) for 48 weeks	Juvenile (4–5 weeks) C57BL/6J mice (male and female)	Carcinomas: 2.5% at week 48	[167]
CDHF diet	High-fat (~24% fat, lard and soybean oil, *w/w*) and choline-deficient diet for 48 weeks		Carcinomas: 25% at week 48	
CDAHF diet	High-fat (~35% fat, *w/w*), choline-deficient, L-amino acid-defined, 0.1% methionine diet for 12, 24, 36, 48 or 60 weeks	Juvenile (5 weeks) C57BL/6J mice (male)	Adenomas: 0% at week 12 and 24; 67% at week 36; 100% at weeks 48 and 60 Carcinomas: 0% at week 12 and 24; 17% at week 36; 9% at week 48; 26% at week 60	[168]
CDA diet	Choline-deficient L-amino acid-defined diet for 24 or 36 weeks	Juvenile to adult (6–8 weeks) C57BL/6 mice	Carcinomas: 30–40% at weeks 24 and 36	[169]
CDA diet and CCl_4_	Choline-deficient L-amino acid-defined diet for 24 or 36 weeks CCl_4_: Multiple i.p. 0.2 mL/kg b.w. for 24 or 36 weeks (1×/week)		Carcinomas: 40% at weeks 24; 100% at week 36	
STAM	STZ: Single s.c. 200 µg (day 2) High fat (32%) diet for 20 weeks	Infant (2 days) C57BL/6J mice (male)	Carcinomas: 100% at weeks 14 to 20	[170]
*Pten* null	-	*Pten* null mice (male and female)	Adenomas: 47% at week 44 and 100% at weeks 74–78; Carcinomas: 66% at weeks 74–78	[171]
MUP-uPA transgenic	High-fat (59% kcal from fat) for 32 or 40 weeks	MUP-uPA mice	Adenomas: 50% at week 32 and 71.4% at week 40; Carcinomas: 16.6% at week 32 and 50% at week 40	[172]

b.wt.: body weight; i.p.: intraperitoneal; s.c.: subcutaneous. HS: high sugar; HF: high fat; CCl_4_: carbon tetrachloride; DEN: diethylnitrosamine; CDHF: choline-deficient high fat; CDA: choline-deficient L-amino acid-defined; CDAHF: choline-deficient L-amino acid-defined high fat; STAM: Stelic Animal Model; STZ: streptozotocin; uPA: urokinase plasminogen activator.

**Table 4:** Summary of the ALD-induced hepatocarcinogenesis protocols in rodents.

Model	Procedure	Animal (Species, Strain, Age)	Timepoints and Incidence of Lesions	References
Ethanol	Liquid low (1%, *w/w*) or high (3%, *w/w*) ethanol diet for ~100–110 weeks	Juvenile to adult (6–7 weeks) Sprague-Dawley rats (male and female)	Neoplastic lesions: 2% at weeks ~100–110 (for both doses and sexes)	[178]
	Ethanol in drinking water 2.5% or 5% (*v/v*) for 104 weeks	Juvenile (4 weeks) B6C3F1 mice (male)	Neoplastic lesions: 34% (2.5%) and 52% (5%) at week 108 Adenomas: 25.5% (2.5%) and 39.6% (5%) at week 108	[179]
MeIQx and Ethanol	MeIQx: 200 mg/kg diet for 8 weeks Ethanol in drinking water 10 or 20% (*v/v*) for 16 weeks	Juvenile (3 weeks) F344/DuCrj rats (male)	Adenomas: ~80% (10%) or ~100% (20%) at week 27 Carcinomas: ~20% (10%) or ~50% (20%) at week 27	[183]
Resistant hepatocyte and Ethanol	DEN: Single i.p. 200 mg/kg b.w.,at week 6 2-AAF: 200 mg/kg in diet for 3 weeks PH: at week 9 Ethanol in drinking water 5% (*v/v*) for 5 or 15 weeks	Juvenile (4 weeks) Wistar rats (male)	Preneoplastic foci: 63%–100% at week, 18 44%–100% at week 28 * Adenomas: 75% at week 18, 94% at week 28 * Carcinomas: 0% at week 18, 0% at week 28 *	[184]
DEN and Ethanol	DEN: Single i.p. 200 mg/kg b.w., at week 9 Ethanol in drinking water 5% (*v/v*) for 16 weeks	Adult (9 weeks) WT rats	Adenomas: 8% at week 25 Carcinomas: 0% at week 25	[182]
		Adult (9 weeks) Cx32 dominant-negative transgenic rats	Adenomas: 25% at week 25 Carcinomas: 25% at week 25	
	DEN: Single i.p.10 mg/kg b.w., at week 2 Lieber-Decarli diet (4.9% of ethanol, *v/v*) for 16 weeks	Infant (2 weeks) C57BL/6 mice (male and female)	Eosinophilic foci: 53% at week 23 Adenomas: 60% at week 23 * Carcinomas: 13% at week 23 *	[185]
	DEN: Single i.p. 1 mg/kg b.w. at weeks 3–4 Ethanol in drinking water 5% (*v/v*) for 3 days, followed by 10% (*v/v*) for 3 days and 10/20% (*v/v*) (alternate days) for 8 weeks, during weeks 16 to 24 or 40 to 48	Juvenile (3–4 weeks) B6C3 mice (male)	Neoplastic lesions: 97.5% at week 48 *	[186]
DEN, Ethanol and HF	DEN: Single i.p. 25 mg/kg b.w. at weeks 2 Liquid ethanol diet (gradually increased from 1% to 2% and 3% (*v/v*, throughout 3 weeks), and maintained at 3.5% (*v/v*)) for 18 or 24 weeks.	Infant (2 weeks) C57BL/6 mice (male)	Carcinomas: ~20% at week 18; ~70% at week 24	[187]

* not statistically different from DEN-only control mice. 2-AAF: 2-Acetylaminefluorene; ALD: alcoholic liver disease; b.wt.: body weight; Cx32: connexin 32; i.p.: intraperitoneal. DEN: diethylnitrosamine; MeIQx: 2-amino-3, 8-dimethylimidazo 4,5-f]quinoxaline; PH: patial hepatectomy.

**Table 5:** Summary of some of the genetically-engineered models established in mice.

Model	Genetic Modification	Timepoints and Incidence of Lesions	References
HBV-transgenic	HBx gene	- Carcinomas: 0% at weeks 16 and 24, 50% at 44–52 weeks, 75% at 60–72 weeks	[195]
		- Adenomas: 8.3% at week 24, 57% at week 44, 12% at week 60 - Carcinomas: 8.3% at week 24, 14% at week 44, 76% at week 60	[196]
	HBx, HBsAg, and pre-S gene	- Carcinomas: 100% at week 80	[199]
		- Preneoplastic foci: 25% at weeks 24–28; ~71% at weeks 36–48; ~83% at weeks 52–80; ~57% at weeks 92–136 - Adenomas: 0% at weeks 24–28; 37.5% at weeks 36–48; ~46% at weeks 52–80; ~75% at weeks 92–136 - Carcinomas: 0% at weeks 24–28; 12.5% at weeks 36–48; ~33% at weeks 52–80; ~25% at weeks 92–136	[200]
	pre-S/S gene (rtA181T/sW172mutation)	- Carcinomas: 8.3% at week 72 (HBsAg low), 23.1% at week 72 (HBsAg high)	[201]
HCV-transgenic	Core gene	- Carcinomas: 25.9–30.8% at week 64	[202]
*c-myc* transgenic	*cnsfo-myc* overexpression	- Preneoplastic foci: 50–83% at weeks 48–56; 80–100% at weeks 72–80 - Adenomas: 40–66% at weeks 48–56; 30–100% at weeks 72–80 - Carcinomas: 0–37% at weeks 48–56; 10–65% at weeks 72–80	[203]
		- Adenomas: 0% at week 24; 20% at week 32; 33% at week 40; ~50 to 90% at weeks 48–64 - Carcinomas: 0% at week 32; 8% at week 40; ~30 to 60% to weeks 48–64	[204]
		- Carcinomas: 0% at week 36; ~10% at week 40; 25 to 50% at week 48; 50% at week 48	[205]
*c-myc*/TGF-α transgenic	Double *c-myc*/TGF-α overexpression	- Carcinomas: ~25% at week 12; 50–75% at week 24; ~100% at weeks 32–36	
		- Preneoplastic foci: 40–100% at weeks 32–40 - Adenomas: 40–100% at weeks 32–40 - Carcinomas: 30–100% at weeks 32–40	[203]
E2F-1 transgenic	E2F-1 overexpression	- Preneoplastic foci: 91% at weeks 32–40; 100% at weeks 40–48 - Adenomas: 73% at weeks 32–40; 100% at weeks 40–48 - Carcinomas: 33% at weeks 32–40; 0% at weeks 40–48	[206]
*c-myc*/E2F-1 transgenic	Double *c-myc*/E2F-1 overexpression	- Carcinomas: ~25% at week 24; 100% at week 32	[205]
*Apc* knockout	*Apc* deletion	- Carcinomas: 67% at week 32	[207]
β-catenin/H-ras mutant	Double *Catnb/Hras* overexpression	- Carcinomas: 100% at week 24	[208]
cMyc + shp53 mice	*c-myc* overexpression, p53 downregulation	- Adenomas + Carcinomas: 38% at week 15	[209]

HBV: hepatitis B virus; HCV: hepatitis C virus; TGF-α: transforming-growth factor-α.

**Table 6:** Summary of some of the HTV protocols for hepatocarcinogenesis in mice.

Genes	Plasmids	Strain	Timepoints and/or Lesions	References
*NRasV12* and *myr-AKT*	7.5 μg of myr-AKT1; 7.5 μg N-RasV12; SB transposase (25:1)	C57BL/6	HCC formation and progression after 2 to 4 weeks post-injection	[220]
*c-Myc* and *β-catenin*	10 μg pT3-EF1a-MYC; 10 μg pT3-N90-CTNNB1; 2.5 μg SB13-Luc transposase-encoding vector	C57BL/6	Poorly to moderately differentiated HCC with solid/trabecular pattern, and immunoexpression of CK19 and nuclear β-catenin	[221]
*β-catenin* and *tert* or *pten*	10 μg pT3-N90-CTNNB1; 10 μg pT3-EF1a-Tert or 10 μg pX330-Pten; 2.5 μg SB13-Luc transposase-encoding vector	C57BL/6	Well to moderately differentiated HCC with trabecular pattern, abundant clear cells, and immuno-expression of glutamine synthetase	[221]
*c-Myc* and *axin1*	10 μg pT3-EF1a-MYC; 10 μg pX330-Axin1; 2.5 μg SB13-Luc transposase-encoding vector	C57BL/6	Well to moderately differentiated HCCs with trabecular pattern	[221]
*c-Myc* and *MCL1*	10 μg pT3-EF1α-c-MYC-shLuc; 5 μg pT3-EF1α-Mcl1; SB transposase (25:1)	C57BL/6 FVB/N Balb/C	Liver tumor formation after 5 to 8 weeks post-injection	[222,223]
*c-met* and *axin1*	20 μg pT3-EF1α-c-Met; 40 μg pX330-Axin1.1; 0.8 μg pCVM/SB	FVB/N	HCC burden at 9 to 12 weeks post-injection showing membranous immunoexpression of E-Cadherin and absence of glutamine synthetase	[224]
*c-Myc* and *myr-AKT*	16–36 µg of mixed plasmids: pT3-EF1a-myrAKT-HA; pT3-EF1α-c-MYC; SB13 transposase-expression plasmid	C57BL/6J	Well to moderately differentiated HCC at 8 to 10 weeks post-injection showing trabecular or nest-like patterns	[225]
*myr-AKT* and/or *Hras*	16–43 µg of mixed plasmids: pT3-EF1a-myrAKT-HA; cDNA fragments of FLAG-HRASV12; SB13 transposase-expression plasmid	C57BL/6J	*Akt* or *Hras*: multiple HCC associated with lipid accumulation after 20–28 weeks post-injection *Akt* and *Hras:* HCC after 8 weeks post-injection with a higher proliferation rate	[226]
*c-met* and *β-catenin*	5 μg pT3-EF5a-hMet-V5; 5 μg pT3-EF5α-S33Y-β-catenin-Myc or 5 μg pT3-EF5α-S45Y-β-catenin-Myc; SB transposase (25:1)	FVB/N	Well-differentiated HCC by 6 to 9.5 weeks post-injection	[227,228,229]
*myr-AKT* and *c-met*	pT3-EF1α -HA-myr-AKT1; pT3-EF1α-V5-c-Met; SB transposase (25:1)	FVB/N	Lethal burden of HCC within 6 to 8 weeks post-injection showing admixture of clear, lipid-rich and lipid-poor, basophilic cells	[229]
*myr-AKT* and *β-catenin*	pT3-EF5-AKT; pT3-EF1α-ΔN90-β-catenin.	FVB/N C57BL/6	Progression to HCC only in vivo passage of steatotic tumor cells from hepatocellular adenomas	[228]

**Table 7:** Advantages, disadvantages and applications of commonly used liver-based in vitro models in liver cancer research.

In Vivo Model	Advantages	Disadvantages	Applications	References
**PHH monolayer**	- Similar to in vivo phenotype - High biotransformation capacity	- High donor-to-donor variability - Progressive dedifferentiation - Fail to represent complex in vivo environment	- Biotransformation studies - Toxicity studies - Drug-induced liver injury studies - Studies related to initiation and promotion of HCC - Liver disease studies	[252,253,254,255,256,257,264,266,271]
**PHH sandwich cultures**	- Prolonged viability - Retained morphology	- Altered protein expression over time - High donor-to-donor variability	- Studies of HCC initiation by carcinogens - Drug-induced liver injury studies	[252,261,268,272,273]
**Liver cell lines monolayer**	- Easy to use - Stable phenotype - Reproducible - Fit for high-throughput and high-content analyses - Allow genetic manipulation - Low cost	- Fail to represent intertumor and intratumor diversity - Less differentiated than PHH - Reduced or absent biotransformation capacity - Fail to represent complex in vivo environment	- Drug-screening - Safety testing - Genotoxicity studies - HCC biology studies - Studies assessing molecular and (epi)genetic modifications in HCC - Overexpression/silencing studies	[253,254,256,274,275,276,277,278,279,280]
**Co-culture models**	- Closer resemblance to in vivo cell heterogeneity and tumor-specific micro-environment - More pathologically relevant model by allowing cell-cell interactions	- Lack of standard protocols - High interlaboratory variability	- Studying influences of non-parenchymal cells and micro-environments on HCC	[253,280,281,282,283,284,285]
**Stem cell-derived model**	- High culture stability - Expandable - Reproducible - Metabolism that resembles PHH	- Lack of standard protocols for isolation of primary CSC	- HCC therapy studies - Studies related to initiation, promotion and drug resistance of HCC	[286,287,288]
**Spheroid models**	- Retained morphology and phenotypic functions - Higher biotransformation capacity compared 2D culture - Display oxygen and nutrient gradients - Closer resemblance to in vivo tumor-specific micro-environment - Closer resemblance to in vivo cell heterogeneity when combining co-culture techniques with spheroid models	- High donor-to-donor variability of PHH - More time-consuming and expensive than 2D culture - Lacks uniformity depending on spheroid-forming method - Low throughput depending on spheroid-forming method - Long-term culture is difficult	- Liver function studies - (Geno)toxicity studies - Liver disease studies - Drug delivery and efficacy studies - Studies investigating role of stem cells - Tumor-growth studies - Angiogenesis studies - Immunotherapy studies	[226,253,254,267,268,289,290,291,292,293,294,295,296]
**Organoid model**	- Mimic functionality and architecture of native tissue - Fit for high-throughput analyses - Expandable - Cryopreservation is possible - Allow genetic manipulation - Retention of tumor heterogeneity - Limited starting material is required - Based on healthy or tumorigenic material	- High cost - Time-consuming - Average success rate	- Regenerative medicine - Personalized drug discovery - Toxicity studies - Gene therapy studies - HBV-related carcinogenesis - Model liver cancer initiation - Molecular and cellular characterization of HCC - Study of inter- and intratumor diversity in HCC	[220,280,284,297,298,299,300,301,302,303]
**Precision-cut-liver slices**	- Capture complex in vivo micro-environment - Retained polarized morphology - Low cost - Automation possible - Closely resemble in vivo gene expression - Based on healthy or tumorigenic material	- Less suitable for high-throughput analyses - Labor-intensive preparation and incubation - Variable culture conditions between studies jeopardizing reproducibility - Reduced albumin and cytochrome P450 expression over time - Technically difficult - Limited availability	- Immunological studies - Toxicity studies - Genotoxicity assessment - Drug-screening - Liver disease studies	[266,288,304,305,306,307,308,309,310,311,312,313,314,315,316]

**Table 8:** Overview of commonly used human liver cancer cell lines.

Cell Line	Cancer Type	HBV/HCV	Gender	Age	Race	References
**HepG2**	Hepatoblastoma	-/-	Male	15	African	[324,337,338]
**Huh-7**	HCC	-/-	Male	57	Asian	[324,337]
**Hep3B**	HCC	+/-	Male	8	African-American	[324,337]
**HepaRG**	HCC	-/+	Female	/	European	[324,333,337]
**MHCC97**	HCC	+/unknown	Male	39	Asian	[324,337,339]
**PLC/PRF/5**	HCC	+/-	Male	24	African	[324]
**SK-HEP-1**	Adenocarcinoma	-/unknown	male	52	European	[324,340]
**SNU-475**	HCC	+/-	Male	43	Asian	[324]
**SNU-423**	HCC	+/-	Male	40	Asian	[324]
**SNU-449**	HCC	+/-	Male	52	Asian	[324]
**C3A**	Hepatoblastoma	-/-	Male	15	European	[277]
**SNU-387**	HCC	+/-	Female	41	Asian	[324]

**Table 9:** Overview of commonly used immortalized human liver cell lines.

Cell Line	Immortalization Method	Gender	Age	Race	References
**Fa2N-4**	SV40 large T antigen Transfection	Female	12	Unknown	[317,341]
**NeHepLxHT**	hTERT Retroviral vector	Male	<1 month	Unknown	[337,342]
**THLE-2**	SV40 large T antigen Retroviral vector	Male	Adult	Unknown	[343]
**PH5CH**	SV40 large T antigen Lipid mediated gene transfer	Male	58	Unknown	[317,344]
